# Antenatal maternal anaemia and infant brain structure: high-field (3 T) and ultra-low-field (64 mT) MRI findings from South Africa

**DOI:** 10.1093/braincomms/fcag318

**Published:** 2026-09-08

**Authors:** Jessica E Ringshaw, Michal R Zieff, Niall J Bourke, Chiara Casella, Layla E Bradford, Simone R Williams, Donna Herr, Marlie Miles, Carly Bennallick, Khula South Africa Data Collection Team, Lauren Davel, Tracy Pan, Tembeka Mhlakwaphalwa, Bokang Methola, Khanyisa Nkubungu, Candice Knipe, Zamazimba Madi, Nwabisa Mlandu, Ringie Gulwa, Pamela Madikane, Sean Deoni, Jonathan O’Muircheartaigh, Dan J Stein, Daniel C Alexander, Derek K Jones, Steven C R Williams, Kirsten A Donald

**Affiliations:** Department of Paediatrics and Child Health, Red Cross War Memorial Children’s Hospital, University of Cape Town, Cape Town, 7700, South Africa; Neuroscience Institute, University of Cape Town, Cape Town, 7925, South Africa; Centre for Neuroimaging Sciences, Department of Neuroimaging, King's College London, London, England, UK; Department of Paediatrics and Child Health, Red Cross War Memorial Children’s Hospital, University of Cape Town, Cape Town, 7700, South Africa; Centre for Neuroimaging Sciences, Department of Neuroimaging, King's College London, London, England, UK; Research Department of Early Life Imaging, School of Biomedical Engineering and Imaging Sciences, King’s College London, London, England, UK; Department for Forensic and Neurodevelopmental Sciences, Institute of Psychiatry, Psychology and Neuroscience, King’s College London, London, England, UK; Department of Paediatrics and Child Health, Red Cross War Memorial Children’s Hospital, University of Cape Town, Cape Town, 7700, South Africa; Neuroscience Institute, University of Cape Town, Cape Town, 7925, South Africa; Department of Paediatrics and Child Health, Red Cross War Memorial Children’s Hospital, University of Cape Town, Cape Town, 7700, South Africa; Neuroscience Institute, University of Cape Town, Cape Town, 7925, South Africa; Department of Paediatrics and Child Health, Red Cross War Memorial Children’s Hospital, University of Cape Town, Cape Town, 7700, South Africa; Department of Paediatrics and Child Health, Red Cross War Memorial Children’s Hospital, University of Cape Town, Cape Town, 7700, South Africa; Neuroscience Institute, University of Cape Town, Cape Town, 7925, South Africa; Centre for Neuroimaging Sciences, Department of Neuroimaging, King's College London, London, England, UK; Maternal, Newborn, Child Nutrition and Health (MNCH) Discovery and Translational (D&T) Sciences Program, Gates Foundation, Seattle, USA; Research Department of Early Life Imaging, School of Biomedical Engineering and Imaging Sciences, King’s College London, London, England, UK; Department for Forensic and Neurodevelopmental Sciences, Institute of Psychiatry, Psychology and Neuroscience, King’s College London, London, England, UK; Medical Research Council (MRC) Centre for Neurodevelopmental Disorders, King’s College London, London, England, UK; Neuroscience Institute, University of Cape Town, Cape Town, 7925, South Africa; South African Medical Research Council (SAMRC), Unit on Risk and Resilience in Mental Disorders, Department of Psychiatry, University of Cape Town, Cape Town, 7925, South Africa; Hawkes Institute and Department of Computer Science, University College London, London, England, UK; Cardiff University Brain Imaging Research Centre (CUBRIC), School of Psychology, Cardiff University, Cardiff, Wales, UK; Centre for Neuroimaging Sciences, Department of Neuroimaging, King's College London, London, England, UK; Department of Paediatrics and Child Health, Red Cross War Memorial Children’s Hospital, University of Cape Town, Cape Town, 7700, South Africa; Neuroscience Institute, University of Cape Town, Cape Town, 7925, South Africa

**Keywords:** neuroimaging, high-field MRI, ultra-low-field MRI, antenatal maternal anaemia, child brain structure

## Abstract

With the evolution of ultra-low-field MRI and the recognition of antenatal maternal anaemia as an important driver of altered neurodevelopment in toddlers and children, it is critical to determine whether these effects are detectable at ultra-low-field (64 mT) in infancy. The aim of this study was to assess the impact of antenatal maternal anaemia on infant brain structure across the first 2 years of life, using high-field (3 T) and ultra-low-field (64 mT) MRI. This neuroimaging substudy was embedded within Khula, an observational population-based birth cohort in South Africa. Pregnant women were enrolled antenatally and postnatally. Mother-child dyads (*n* = 394) were followed prospectively with a subsample attending neuroimaging at ∼3, 6, 12, 18 and 24 months of age. Anaemia was classified using World Health Organization thresholds, and neuroimaging data were processed using MiniMORPH. Linear mixed-effects models were used to investigate associations between antenatal maternal anaemia status and absolute regional infant brain volumes using high-field and ultra-low-field MRI. In repeated measures high-field (*n* = 195) and ultra-low-field (*n* = 341) infant neuroimaging subsamples, the prevalence of antenatal maternal anaemia was 28.24% (37/131) and 29.76% (61/205), respectively. Maternal anaemia in pregnancy was associated with altered child brain structure across both MRI systems, with group differences becoming detectable at ∼12 months. In the ultra-low-field subsample, infants born to anaemic mothers had 3.77% smaller intracranial volume (β = −0.24, *P* = 0.004) and 3.32% smaller putamen volumes (β = −0.18, *P* = 0.040) across the first 2 years of life. The interaction between antenatal maternal anaemia and age was significant for the caudate nucleus (β = −0.13, *P* = 0.038) and corpus callosum (β = −0.15, *P* = 0.007). Antenatal maternal anaemia was associated with 3.70% and 4.29% smaller caudate nucleus volumes at 18 and 24 months of age, respectively. Similarly, infants born to anaemic mothers had 4.39% smaller corpus callosum volumes by 12 months and 6.27% smaller corpus callosum volumes by 24 months. Postnatal child anaemia and antenatal maternal iron deficiency status were not associated with total or regional child brain volumes in the ultra-low-field subsample from this cohort. Maternal anaemia remained a robust predictor of volume differences in sensitivity analyses. This study is the first to demonstrate that the impact of maternal anaemia in pregnancy on child brain structure is detectable as early as infancy. The implications of this research are 2-fold: (i) informing the feasibility of ultra-low-field MRI in low- and middle-income countries and (ii) the timing and optimization of targeted recommendations for anaemia management in practice and policy.

## Teaser

Ringshaw *et al.* demonstrated that antenatal maternal anaemia may impact child brain structure as early as infancy, with detectable effects from 12 months of age, using ultra-low-field MRI. This research informs the feasibility of ultra-low-field MRI in low- and middle-income countries and the optimization of anaemia prevention and intervention strategies.

## Graphical Abstract

**Graphical Abstract:**
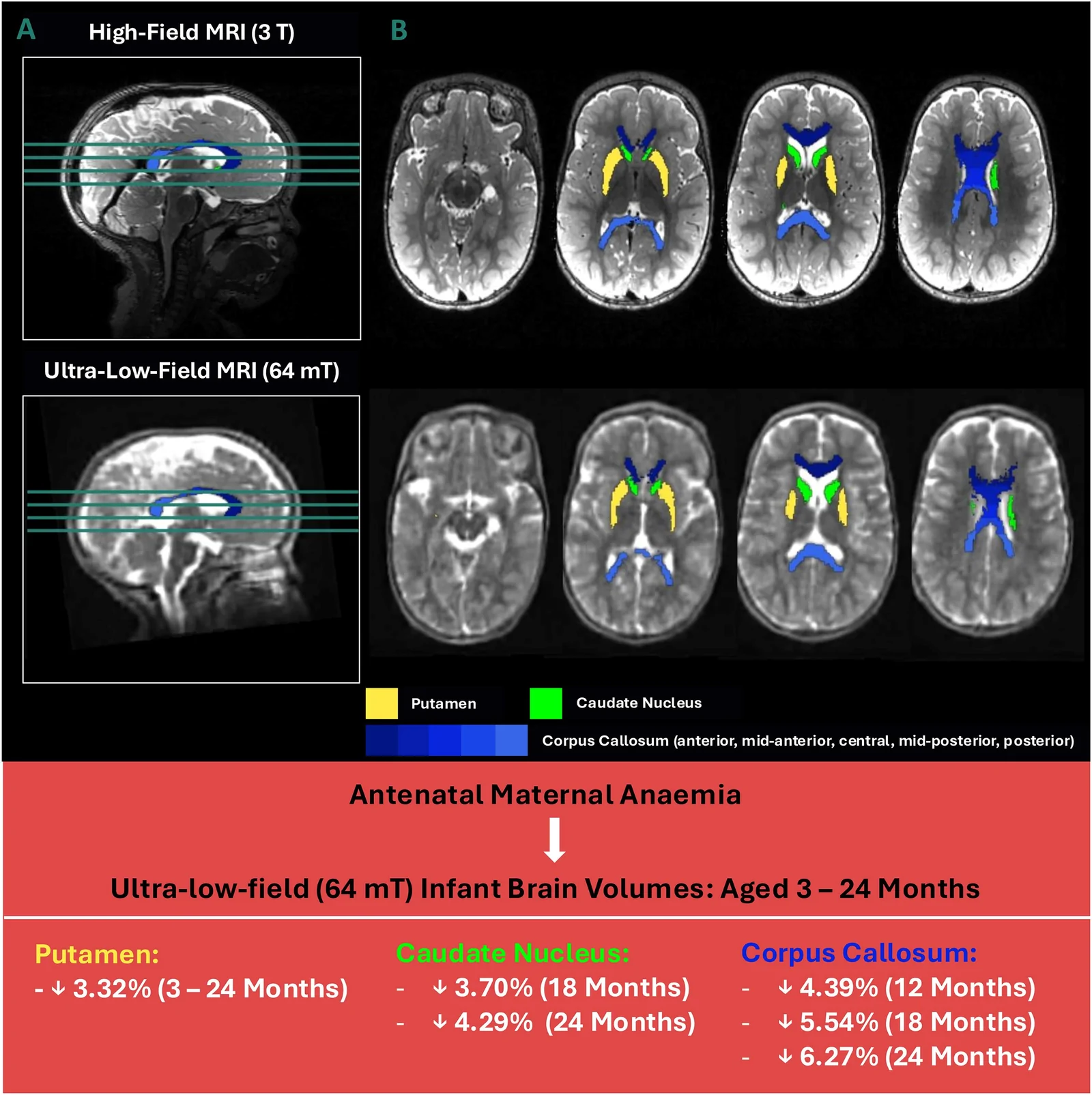

## Introduction

Antenatal maternal anaemia, characterized by low serum haemoglobin during pregnancy, affects more than one-third (∼36% as of 2023) of expecting women worldwide.^1-3^ Based on the most recent World Health Organization (WHO) report published in 2025,^3^ efforts to reduce the prevalence of anaemia in women of reproductive age have stagnated over the last two decades, with most countries showing little to no progress or even a deterioration. Consequently, projections from current trends (2012–23) suggest that the world is not on track to achieve the 2030 nutrition target of a 50% reduction in the prevalence of anaemia among this population group. While this pattern is observed globally, the burden of disease is unevenly distributed, with a much higher prevalence in low- and middle-income countries (LMICs), particularly in sub-Saharan Africa and South-East Asia, which together account for 60% of the global burden among women of reproductive age.^2,3^ This is largely driven by risk factors such as malnutrition, infectious diseases and alcohol use.^4,5^ The impact of the COVID-19 pandemic is likely to have exacerbated this public health problem by disrupting health, educational and social protection systems while simultaneously increasing the risk of food insecurity.^1,6^ With growing evidence for the foetal origins of brain health and a renewed focus on maternal health in pregnancy,^7^ addressing antenatal maternal anaemia is emerging as a priority for strategies aimed at improving child development.^8^

While the aetiology of anaemia is multifaceted, chronic iron deficiency is known to be the most common cause due to its limiting impact on haemoglobin production.^9^ This negatively affects the haemoglobin-facilitated delivery of oxygenated blood to vital metabolic organs, including the foetal brain during pregnancy.^10,11^ Recent epidemiological research from the Khula South Africa study^12^ has demonstrated that the prevalence of maternal iron deficiency in pregnancy may be as high as 50% after adjustment for inflammation, accounting for half of all anaemia cases in this cohort.^13^ In turn, maternal anaemia and iron deficiency in pregnancy have consistently been identified as risk factors for poor birth^14,15^ and child developmental outcomes,^16,17^ which may have a long-term impact on the next generation’s health, cognition and behaviour.^1^ This is reiterated by a South African study suggesting that maternal anaemia may be one of the main drivers for children failing to reach their developmental potential.^7^

Thus far, a few MRI studies have suggested that the developing brain may be particularly sensitive to maternal anaemia and iron deficiency in pregnancy,^18-20^ but the results have been largely inconsistent due to widely varying research methods. To address this, a neuroimaging substudy of the Drakenstein Child Health Study (DCHS)^21^ in South Africa compared the relative effects of antenatal maternal and postnatal child anaemia on child brain structure.^8,22^ Maternal anaemia in pregnancy was associated with smaller volumes of the corpus callosum, putamen and caudate nucleus in toddlers (aged 2–3 years)^8^ and school-age children (aged 6–7 years)^22^ in this cohort. These findings suggest that the effects of antenatal maternal anaemia on child brain structure may be regionally consistent and persist with age, reinforcing the importance of optimizing anaemia interventions in women of reproductive age before and during pregnancy.^8,22^

Despite this emerging evidence, further corroboratory neuroimaging research is required to assess whether the effects of antenatal maternal anaemia on child brain structure can be generalized to other community settings and, critically, whether they can be detected in infancy. Therefore, the primary aim of this study was to investigate the impact of antenatal maternal anaemia on child brain structure in South African children across the first 2 years of life using conventional high-field (HF; 3 T) MRI as proof of concept. The secondary aim of this research was to replicate the HF analyses using ultra-low-field (ULF; 64 mT) MRI, a quickly evolving field with the potential to increase neuroimaging accessibility at scale in LMICs. The novel Hyperfine Inc., Swoop® MRI scanner is more cost-effective, more power-efficient and much less noisy than conventional systems, allowing it to be easily integrated into low-resource clinical settings for paediatric use.^23^ Recent cross-validation research using paired data from the Khula cohort in South Africa has demonstrated high concordance between 3 T and 64 mT volume estimates for regions of interest known to be associated with maternal anaemia.^24^ While this supports the meaningful interpretation of ULF data in anaemia research, the utility of this system in detecting group differences in regional brain volumes associated with anaemia is largely unknown. Therefore, this research aimed to play a key role in assessing this novel system’s comparative ability to answer clinical questions about antenatal maternal anaemia as detected by conventional MRI in South Africa.

With larger sample sizes and longitudinal postnatal data, the Khula ULF substudy was also well-positioned to delineate the timing of effects by assessing the relative contributions of antenatal maternal anaemia versus postnatal child anaemia on infant brain structure. In parallel, the role of iron deficiency was explored as a potential underlying neurobiological driver of associations between anaemia and regional infant brain volumes. Overall, this research aimed to validate the feasibility of ULF MRI by answering these important clinical questions, while also aiming to inform the timing and optimization of more targeted recommendations for anaemia management in both practice and policy.

## Materials and methods

### Ethics statement

The Khula South Africa birth cohort study received ethical approval from the University of Cape Town Human Research Ethics Committee (666/2021; 782/2022). All mothers provided written informed consent for cohort participation at Khula enrolment and for their children to participate in study-specific procedures across timepoints. Given the longitudinal nature of the cohort, study consent is obtained from mothers annually.

### Study design and setting

This research is embedded within Khula South Africa, an observational population-based birth cohort study in Gugulethu, an urban informal settlement in the Western Cape Province of South Africa.^12^ The overarching purpose of this longitudinal study was to characterize executive functioning in the first 1000 days of life, explore the underlying neural pathways in the developing brain and investigate the role of known risk factors through the use of multimodal tools including MRI. The aim of this neuroimaging substudy was to use HF (3 T) and ULF (64 mT) MRI data to investigate the associations between anaemia, iron deficiency and child brain structure within the first 2 years of life (3–24 months).

Mother-child dyads were recruited antenatally from the Gugulethu Midwife Obstetrics Unit and postnatally from clinics in the surrounding area. Previous research from the Khula South Africa cohort^13^ has demonstrated that ∼65% of mothers are unemployed, with 63% of households earning a total income of less than 5000 ZAR per month (<300 USD). For context, the cost of a grocery shop consisting of food staples (2 L milk, 1 loaf of brown bread, 1 kg brown sugar, 1 kg potatoes, 2.5 kg flour and 1 kg chicken) was ∼300 ZAR (20 USD), as of March 2026. Food insecurity was reported by >50% of the mothers, and the prevalence of antenatal maternal anaemia was 35%, with approximately half of cases being attributed to iron deficiency after adjustment for inflammation.^13^ Maternal HIV was associated with an increased risk of anaemia in pregnancy, highlighting the relevance of infection as a key aetiological factor for consideration. The demographic and anaemia profile of this cohort is comparable to statistics from the National Department of Health’s Demographic and Health Survey,^25^ making this study community representative of the South African population.

Khula mother-child dyads were followed prospectively with a subsample attending neuroimaging study visits at ∼3, 6, 12, 18 and 24 months of age.

### Participants

**Figure 1:**
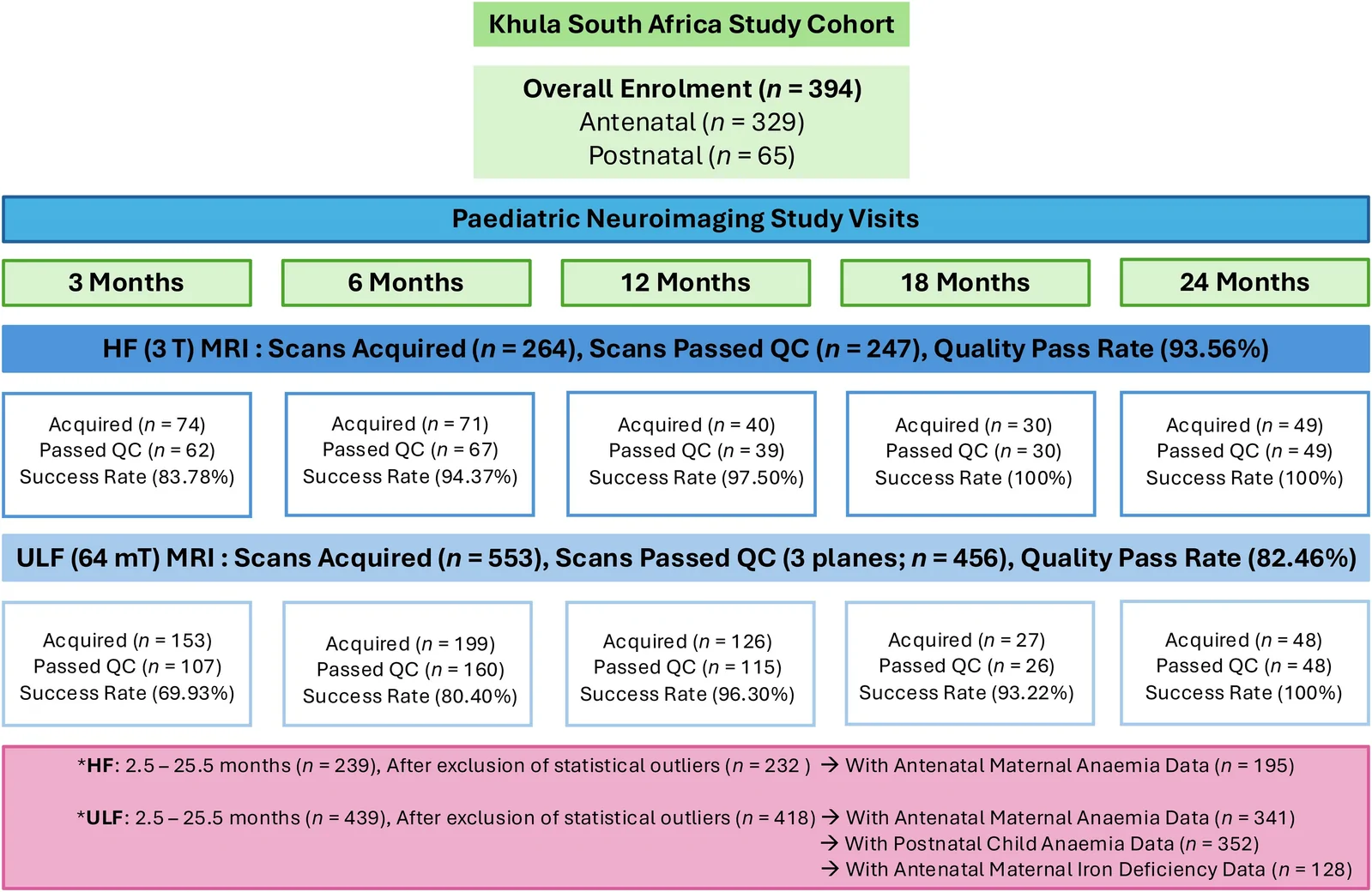
**Study flowchart demonstrating the acquisition and quality pass rates of HF (3 T) and ULF (64 mT) neuroimaging data across study visits and the final sample size determination with corresponding antenatal maternal anaemia data.** All neuroimaging sample sizes represented include repeated measures. The primary analysis assessing the impact of antenatal maternal anaemia on child brain structure was conducted separately using both HF and ULF subsamples. The analyses assessing the effects of postnatal child anaemia (secondary analysis) and antenatal maternal iron deficiency (tertiary analysis) on child brain structure were only conducted in the larger ULF subsample due to increased power for the detection of effects.

In South Africa, women were recruited (December 2021–November 2022) if they met the eligibility criteria of being over the age of 18 years and in the third trimester of pregnancy (28–40 weeks of gestation) or up to 3 months postpartum. Exclusion criteria included multiple pregnancies, psychotropic drug use during pregnancy, infant congenital malformations or abnormalities (e.g. spina bifida, Down syndrome) and severe birth complications (e.g. uterine rupture, birth asphyxia). Overall, a total of 394 mother-child pairs from South Africa were enrolled in the Khula study, with 329 women retained from antenatal recruitment and 65 women retained from postnatal recruitment (Fig. 1; Study flowchart).

Of these eligible Khula babies in South Africa, 264 T2-weighted (T2W) HF scans were acquired across study visits, of which 247 passed quality check (QC) procedures (93.56%). Similarly, 553 ULF T2W scans (complete with axial, coronal and sagittal planes) were collected across timepoints with 456 (82.46%) passing QC. The neuroimaging subsamples for HF and ULF analyses were compiled for children between 3 and 24 months of age (with a 2-week window on either side; 2.5–25.5 month range) with useable brain scans (passed QC) across timepoints. Statistical outliers were excluded if extreme (*z*-scores above and below 3 SD from the sample mean) or moderate (above and below 2 SD from the sample mean) but consistent across multiple regions of interest (ROIs; 3 for HF to maximize sample size and power; 2 for ULF due to increased risk of measurement error with the ULF system). The final repeated measures subsamples for HF (232 scans; 156 unique infants) and ULF (418 scans; 249 unique infants) datasets included an overlap of 132 unique infants with eligible scans acquired on both MRI systems.

**Figure 2:**
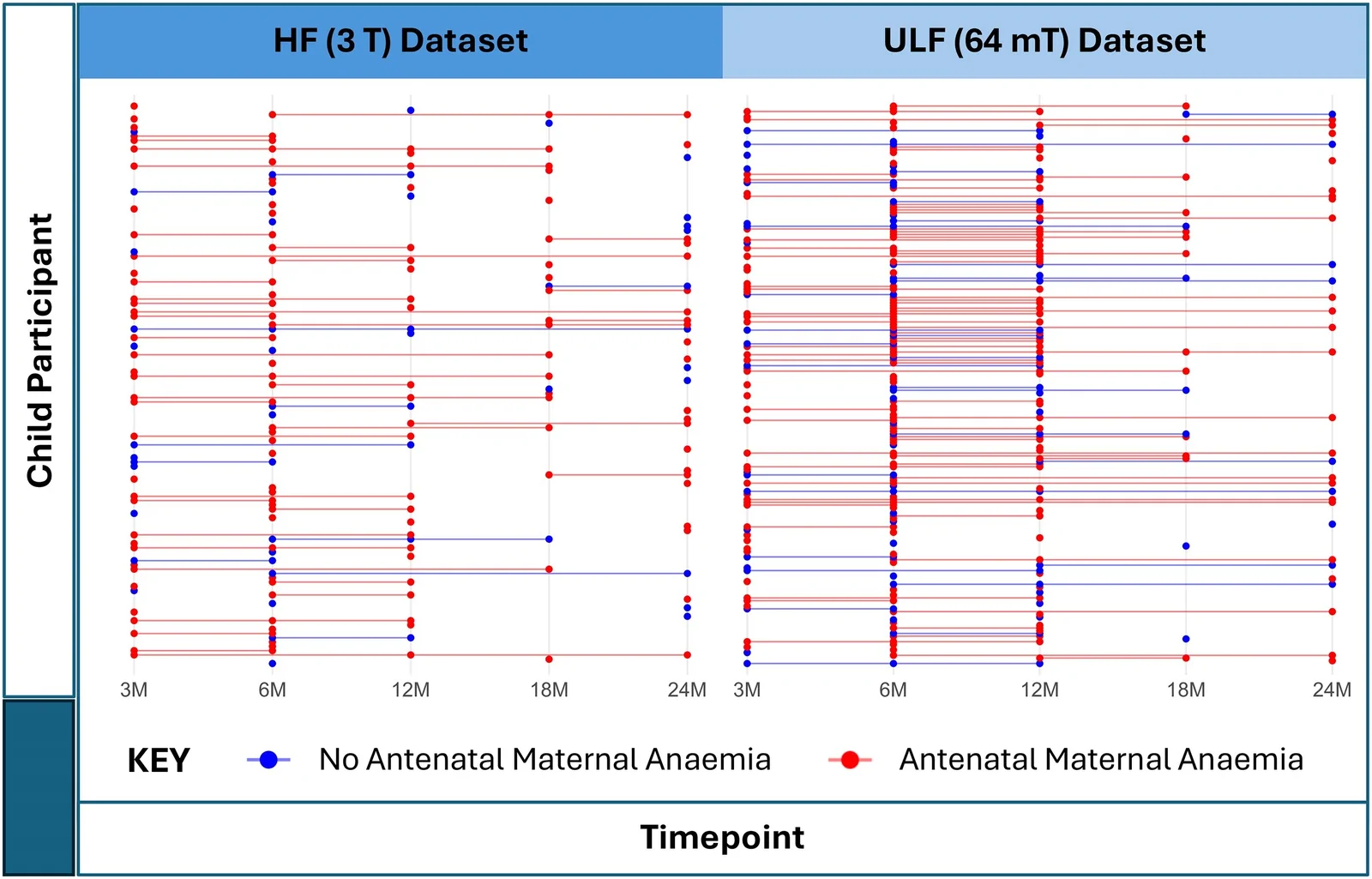
**Repeated measures data availability for participants across study timepoints for HF (3 T; left) and ULF (64 mT; right) datasets.** Each datapoint reflects a scan acquired for the HF subsample (left) or ULF subsample (right). The lines between datapoints reflect where longitudinal data are available for the same participant across study timepoints. For the primary analysis with available maternal anaemia data, the final samples for HF (195 repeated measures scans; 131 unique infants) and ULF (341 repeated measures scans; 205 unique infants) datasets included an overlap of unique children that were scanned on both MRI systems (*n* = 112). Timepoints refer to study visits at 3 months (3 M), 6 months (6 M), 12 months (12 M), 18 months (18 M) and 24 months (24 M). In the HF subsample (*n* = 195 repeated measures scans; *n* = 131 unique infant subjects), 80/131 (61.10%) of babies only had MRI data available at a single timepoint. Repeated measures data were available for 51/131 (38.93%) babies across 2 timepoints (40/131; 30.53%), 3 timepoints (9/131; 6.87%) and 4 timepoints (2/131; 1.53%). In the ULF subsample (*n* = 341 repeated measures scans, *n* = 205 unique infant subjects), 101/205 (49.27%) of babies only had MRI data available at a single timepoint. Repeated measures data were available for 104/205 (50.73%) babies across 2 timepoints (75/205; 36.59%), 3 timepoints (26/205; 12.68%) and 4 timepoints (3/205; 1.46%).

For the primary analysis (Fig. 1; Fig. 2), the repeated measures neuroimaging subsamples with corresponding antenatal maternal anaemia data included 195 HF scans (131 unique infants) and 341 ULF scans (205 unique infants). The overlap of unique children with maternal anaemia data and eligible scans on both HF and ULF systems was 112. Similarly, the ULF neuroimaging subsample (Fig. 1) with repeated measures for infants with corresponding postnatal child anaemia data (secondary analysis) included 352 scans (214 unique infants), and corresponding antenatal maternal iron deficiency (tertiary analysis) included 128 scans (73 unique infants). All sample sizes include repeated measures data available across study timepoints (Fig. 2).

### Measures

#### Contextual measures

Sample characteristics were acquired for demographic [age, sex, socio-economic status (maternal education, total household income, maternal employment)] and antenatal maternal health (maternal HIV status, maternal depression, prenatal alcohol exposure, prenatal tobacco exposure) variables. These contextual data were previously described for the full cohort^13^ and were included for use as covariates in this neuroimaging substudy.

#### Anaemia status

Antenatal maternal anaemia status was assessed based on the lowest serum haemoglobin concentration and corresponding gestational age available from a full blood count acquired at Khula study enrolment in pregnancy and/or extracted from antenatal medical records (National Health Laboratory Services). For mothers with no recorded gestational age data, haemoglobin measurements were assigned to trimester 3 based on likely time of recruitment and highest risk coinciding at this timepoint. Based on the 2024 WHO guidelines, antenatal maternal anaemia status was dichotomously classified using thresholds of <11 g/dL in trimesters 1 and 3 and <10.5 g/dL in trimester 2.^26^ Further classification based on anaemia severity was defined as mild (haemoglobin 10.0–10.9 g/dL in trimesters 1 and 3; 9.5–10.4 g/dL in trimester 2), moderate (haemoglobin 7.0–9.9 g/dL in trimesters 1 and 3; 7–9.4 g/dL in trimester 2) and severe (haemoglobin <7.0 g/dL) anaemia.

Haemoglobin was measured for infants at each study visit (3, 6, 12, 18 and 24 months). Dichotomous classifications for postnatal child anaemia were determined for each infant using age-specific cut-offs for haemoglobin based on WHO guidelines^27^ for children over 6 months and local guidelines for children under 6 months. These thresholds have previously been described for this cohort.^13^ An overall classification for child anaemia status was determined based on the diagnosis of anaemia at least once across study visits. This was used for the assessment of group differences in sample characteristics between infants who had ever been anaemic versus infants who had never been anaemic between 3 and 24 months of age. Minimum child haemoglobin and corresponding age across study visits were also determined for each infant and reported across both groups. For modelling with the repeated measures subsample (multiple scans per child in some instances), child anaemia status was assessed based on the most severe presentation of anaemia as indicated by the minimum haemoglobin measure observed between birth and the time of scan.

#### Iron deficiency status

Iron deficiency status was assessed using serum ferritin with adjustment for inflammation based on inflammatory biomarkers [highly sensitive C-reactive protein (*hs*CRP) and alpha-1-acid glycoprotein (AGP)]. This was conducted using BRINDA (Biomarkers Reflecting Inflammation and Nutritional Determinants of Anaemia),^28^ a regression modelling approach^29^ that corrects continuous serum ferritin concentrations based on *hs*CRP and AGP concentrations. As previously described in this cohort,^13^ iron deficiency status was dichotomously classified using standard WHO thresholds of <12 and <15 μg/L for adjusted serum ferritin in infants and women, respectively.^30^

#### Neuroimaging outcomes

##### Scan acquisition

Both HF and ULF MRI were acquired at the Cape Universities Body Imaging Centre. HF MRI was conducted on a 3 Tesla (3 T) Siemens Skyra scanner using a 16- or 32- channel head coil for infants between 3–12 and 12–18 months of age, respectively. ULF MRI was conducted using the Hyperfine Swoop® Inc., 64 mT system. This involved running separate sequences for the acquisition of structural data across axial, coronal and sagittal planes. Structural T2W sequences were chosen for analysis as this sequence is typically more suitable for assessing brain volume in infants^31^ and is currently the best-developed sequence on the novel ULF system.^23^ Neuroimaging data were collected during non-sedated sleep using well-established clinical approaches and strategies for paediatric scanning developed by our team.^24,32^ Neuroimaging success rates across field strengths increased with age from the 3-month to 24-month study visit (Fig. 1). This was largely due to decreased motion and ongoing improvements in the scanner acquisition software by the time we scanned older children, as reported in previous work on this cohort by our team.^24^ The neuroimaging protocols and MRI specifications have been described separately.^24^ All HF scans were subject to radiological review, and infants with incidental findings were referred via established institutional pathways.

##### Neuroimaging processing

All HF and ULF scans were subject to a rigorous QC protocol using a standardized checklist for assessing image quality on Flywheel, a harmonized data management platform.^33^ This was completed by two senior neuroimaging staff members trained to recognize artefacts such as motion and electrical interference. Factors affecting scan success have been reported previously.^24^ Only useable scans that had passed QC (prioritizing ‘good’ quality ratings) were included for analyses (Fig. 1).

Neuroimaging processing was conducted using Docker containers and the gear functionality of Flywheel^33^ for reproducible and version-controlled output. All software developed for the UNITY project has been shared as an open-source resource on GitHub. The first processing step only applied to the ULF data and involved using a multi-resolution registration pipeline to reconstruct orthogonally acquired anisotropic images across axial, coronal and sagittal planes into single 1.5 mm^3^ isotropic images of higher effective resolution than the individual planes.^34^

The second step of processing used the MiniMORPH pipeline (described in full previously)^35^ to segment neuroimaging data from across HF and ULF field strengths. This is a novel approach using age-specific templates derived from ULF Khula data with specified age cut-offs for 3, 6, 12, 18 and 24 months. This was designed to avoid biases associated with traditional paediatric templates based on HF data predominantly collected in North America. Tissue and cerebrospinal fluid priors were generated by registering age-matched T2W images from the Baby Connectome Project (BCP)^36^ to the study-specific templates using SynthMorph,^37^ with the corresponding white matter, grey matter and CSF probability maps transformed accordingly. A tissue prior was created by combining the white matter and grey matter probability maps, and a skull prior was generated by dilating the brain mask to improve classification of non-brain regions. Subcortical grey matter parcellations from the BCP atlas were registered to the 12-month template using ANTs,^38^ manually refined where needed to ensure anatomical accuracy and then propagated to the remaining age-specific templates. Corpus callosum masks were derived from the Penn-CHOP atlas^39^ and registered to the 12-month template. Because the Penn-CHOP atlas only provides a whole-corpus-callosum mask, callosal parcellations were then generated by manually segmenting the corpus callosum in template space according to the Desikan-Killiany subdivisions (anterior, mid-anterior, central, mid-posterior and posterior).^40^ The resulting parcellated callosal mask was reviewed for anatomical accuracy and propagated to the remaining age-specific templates using the same ANTs-based transformation framework.^38^ Ventricle, cerebellum and brainstem masks were manually delineated in template space and reviewed by an expert rater. For each scan, the reconstructed isotropic T2W image was registered to the appropriate age-specific template using ANTs, and the inverse transformations were used to resample tissue priors and anatomical masks into native space. Segmentation into the three primary tissue classes (tissue, CSF and skull) was then performed using ANTs Atropos^38^ with prior probability weighting. To refine the segmentation, the ventricle mask was applied to the CSF class to isolate ventricular CSF from extra-ventricular CSF, the subcortical grey matter mask was applied to extract subcortical structures from the overall tissue class, and the brainstem and cerebellum masks were applied in native space to distinguish these infratentorial regions from supratentorial tissue and CSF. A final native space atlas was completed for each scan with fslmaths,^41^ including all segmented regions in native space. Volume estimates were extracted with fslstats^41^ into csv files for statistical analysis.

### Statistical analysis

#### Sample characteristics

Demographic and clinical data were presented as means and standard deviations for continuous variables and frequencies and percentages for categorical variables. All statistical analyses were conducted in RStudio (2026.01.1+403) for R (version 4.5.2), and a significance level of 0.05 was used throughout. Group differences (based on maternal anaemia status, child anaemia status and maternal iron deficiency status separately) in sociodemographic and maternal exposure variables were calculated using unpaired *t*-tests for continuous data and chi-squared tests or Fisher’s exact tests for categorical data. This was assessed based on the number of unique subjects in each subsample, excluding repeated measures data for children with multiple scans. This approach was used to avoid skewing results with duplicated demographic and clinical observations for the same infants. The only exception to this approach was child age at scan, which was considered for the full repeated measures subsample, with a proportion of children having multiple scans acquired at different ages. Checks for assumptions of normality were conducted throughout.

#### Neuroimaging regions of interest

Given that antenatal maternal anaemia has previously been associated with smaller volumes of the caudate nucleus, putamen and corpus callosum in children at 2–3^8^ and 6–7^22^ years of age in a similar South African birth cohort, these structures were chosen as key ROIs for a hypothesis-driven analysis. Intracranial volume (ICV) was included to investigate group differences in total brain volume. The total caudate nucleus and total putamen were computed as the sum of left and right hemisphere volumes for these basal ganglia structures. The total corpus callosum was computed as the sum of individually segmented posterior, mid-posterior, central, mid-anterior and anterior regions.

#### Primary analysis: antenatal maternal anaemia status

##### Exploratory analyses

As an exploratory step, absolute regional brain volumes were plotted against child age in months at the time of scan for both HF and ULF subsamples. LOESS (locally estimated scatterplot smoothing) curves were used to visually assess changes in growth with age for ICV and each ROI (putamen, caudate nucleus, corpus callosum). The trend of the data was observed to qualitatively classify whether regional increases in absolute brain volumes with age represented a linear relationship or a non-linear relationship.

##### Statistical modelling

The associations between antenatal maternal anaemia status and absolute regional infant brain volumes were assessed across field strengths to compare findings for HF and ULF MRI systems. The HF dataset was used as proof of concept and to attempt to replicate findings from previously published studies. Analyses were repeated on the ULF system to determine whether the same brain volume trajectories and effects of anaemia could be observed and detected at ULF.

Given the repeated measures study design with individual infant data across multiple study visits in both HF and ULF subsamples, linear mixed-effects (LME) models were fitted to account for individual differences in assessing the association between antenatal maternal anaemia status and absolute regional brain volume trajectories. Child sex at birth and child age at scan were included in all models as covariates known to affect child brain structure *a priori.*^42^ Using a data-driven approach, the observed trend of the data in exploratory analyses (LOESS curves) was used to inform the use of age in statistical modelling. More specifically, age in months was entered as an absolute value for brain regions with an observed linear growth trajectory or as a logarithmic transformation [log (age)] for brain regions with an observed non-linear growth trajectory. ICV was not included in the ROI models due to high levels of demonstrated multicollinearity with age at scan based on zero-order correlations in both HF (*r* = 0.814, *P* < 0.001) and ULF (*r* = 0.747, *P* < 0.001) subsamples. Therefore, only scan age was included in models for absolute regional brain volumes, serving as a proxy for ICV. Overall, antenatal maternal anaemia, child age (or the logarithmic transformation of child age at scan) and child sex were entered as fixed effects with a random intercept to account for repeated measures.

Demographic and maternal exposure variables with observed group differences were considered for inclusion in analyses as potential confounders, only if correlated with regional brain volumes in preliminary steps. Visual inspection of the plotted data (LOESS curves in exploratory analyses) informed the inclusion of an interaction for the effect of antenatal maternal anaemia status on absolute regional child brain by age for linear growth trajectories or the logarithmic transformation of age [log (age)] if relevant for non-linear growth trajectories. This was only included as a fixed effect in the ULF models due to a larger sample size (*n* = 341) and greater statistical power in this dataset. If the interaction was not significant in these ULF models, they were re-run without the interaction effect for the assessment of main effects alone. Interactions were excluded from HF analyses due to the smaller sample size (*n* = 195) and the sparse distribution of data across age groups, which may have contributed to unreliable estimates and an increased risk of overfitting.

*Post hoc* testing was conducted on the final HF and ULF LMEs using estimated marginal means (EMMs) for the regression line of best fit. For brain regions with no interaction between antenatal maternal anaemia and age, EMMs were used to calculate the overall adjusted percentage difference in regional brain volumes between groups. For brain regions with a significant interaction between antenatal maternal anaemia status and age (or the logarithmic transformation of age), EMMs were computed for specified age groups (3, 6, 12, 18 and 24 months). Multiple pairwise comparisons were conducted to determine group differences across ages and to identify the age at which the association between antenatal maternal and regional brain volumes became significant. The Holm method was used for multiple comparison correction.^43^

#### Secondary analysis: postnatal child anaemia status

The role of postnatal child anaemia was explored using ULF MRI for maximum statistical power. In these LMEs, child anaemia status was based on the most severe presentation of anaemia observed between birth and the time of scan for each repeated measure. First, the exploratory analyses and models described above for maternal anaemia were replicated with postnatal child anaemia as the primary predictor of interest. This series of models was run to assess the independent associations between child anaemia and regional child brain volumes. Second, the final models from the primary analyses assessing the impact of antenatal maternal anaemia status on regional brain volumes were repeated with the addition of postnatal child anaemia status as a covariate. This assessed the relative contributions of antenatal versus postnatal exposures and examined whether the observed associations between maternal anaemia and regional child brain volumes remained after controlling for child anaemia.

#### Tertiary analysis: iron deficiency status

Given that iron deficiency is the most common cause of anaemia, it was investigated as a potential predictor of child brain structure. Based on this proposed aetiological underpinning, it was hypothesized that the association between iron deficiency status and regional brain volumes would mimic that of anaemia status. Therefore, this tertiary analysis was only run for variables with demonstrated associations between anaemia (antenatal maternal versus postnatal child) and child brain structure in primary and secondary analyses; the role of iron deficiency was only assessed if the effects of anaemia were significant. Given power limitations, LMEs were only used to assess main effects.

#### Sensitivity analyses

Sensitivity analyses for all LME models were conducted to account for the potential confounding role of relevant maternal exposures (with observed group differences in prevalence). Multicollinearity was considered in the establishment of all models.

## Results

### Primary analysis: antenatal maternal anaemia | HF and ULF MRI

#### Sample characteristics: antenatal maternal anaemia status

In the HF neuroimaging subsample (Table 1), 131 mothers [mean (SD) age at enrolment: 29.79 (5.62) years, age range of 19.00–41.70 years] had antenatal maternal haemoglobin data available. Minimum haemoglobin measurements were predominantly acquired in the second [35.88% (47/131)] and third trimester of pregnancy [34.35% (45/131)], at a median (IQR) of 22 (14–32) weeks. Of these mothers, 28.24% (37/131) were classified as anaemic during pregnancy, with 70.27% (26/37) of these cases being mild, 27.03% (10/37) moderate and 2.70% (1/37) severe. With repeated measures available for 38.93% (51/131) of the subsample included (Fig. 2), a total of 195 infant scans [average age at time of scan: 11.91 (6.37) months, age range of 2.56–24.84 months] with corresponding maternal anaemia data were available. There were no differences in maternal characteristics or child characteristics between groups based on antenatal maternal anaemia status (*n* = 131; Table 2), with the exception of household income (*P* = 0.031), which was lower in mothers with anaemia than in non-anaemic mothers.

**Table 1:** Antenatal maternal anaemia profile across HF (3 T) and ULF (64 mT) subsamples

	HF subsample (*n* = 131)^a^			ULF subsample (*n* = 205)^a^		
Variable^a^	Maternal anaemia (*n* = 37)	No maternal anaemia (*n* = 94)	*P*	Maternal anaemia (*n* = 61)	No maternal anaemia (*n* = 144)	*P*
**Antenatal maternal anaemia profile** ^b^						
Severity in pregnancy						
Mild	26 (70.27)	N/A	N/A	41 (67.21)	N/A	N/A
Moderate	10 (27.03)	N/A	N/A	19 (31.15)	N/A	N/A
Severe	1 (2.70)	N.A	N/A	1 (1.64)	N/A	N/A
Gestational age at Hb measurement^d,e^	27.39 (8.22) [7.00–40.00]	20.95 (10.40) [5.00–41.00]	<0.001***	26.21 (9.48) [6.00–39.00]	21.07 (9.90) [5.00–41.00]	<0.001***
Pregnancy trimester Hb measured^c,e^						
First^c^	2 (5.41)	25 (26.60)	0.011*	9 (14.75)	33 (22.92)	0.010*
Second^c^	13 (35.14)	34 (36.17)		18 (29.51)	59 (40.97)	
Third^c^	18 (48.65)	27 (28.72)		29 (47.54)	37 (25.69)	
Trimester Unknown	4 (10.81)	8 (8.51)		5 (8.20)	15 (10.42)	
Minimum Hb in pregnancy (g/dL)						
Trimester 1 (HF: *n* = 27, ULF: *n* = 42)^c^	10.45 (0.64) [10.00–10.90]	12.18 (0.70) [11.00–13.50]	0.002**	10.26 (0.96) [7.90–10.90]	12.12 (0.69) [11.00–13.50)	<0.001***
Trimester 2 (HF: *n* = 47, ULF: *n* = 77)^c,d^	9.78 (0.45) [8.80–10.30]	11.82 (0.84) [10.60–13.80]	<0.001***	9.77 (0.65) [7.7–10.30]	11.68 (0.72) [10.60–13.20)	<0.001***
Trimester 3 (HF: *n* = 45, ULF: *n* = 66)^c,d^	9.54 (1.39) [5.30–10.90]	11.65 (0.62) [11.00–13.50]	<0.001***	9.61 (1.30) [4.40–10.90]	11.64 (0.61) [11.00–13.50)	<0.001***

Abbreviations: HF, high-field; ULF, ultra-low-field; T, tesla; mT, millitesla; Hb, haemoglobin; g, grams; dL, decilitre.

Group differences based on antenatal maternal anaemia status were calculated using unpaired *t*-tests for continuous data and chi-squared tests or Fisher’s exact tests for categorical data.

^a^The total and group subsamples represent the number of unique subjects, excluding repeated measures data for children with multiple scans to avoid duplication of observations for infants with multiple scans. Values for continuous variables are presented as mean (standard deviation) [range]. Values for categorical variables are presented as count (%).

^b^Maternal anaemia during pregnancy was classified according to the WHO threshold of <11 g/dL for trimesters 1 and 3 and <10.5 g/dL for trimester 2. Severity categorizations were made indicating mild (haemoglobin 10.0–10.9 g/dL in trimesters 1 and 3; 9.5–10.4 in trimester 2), moderate (haemoglobin 7.0–9.9 g/dL in trimester 1; 7–9.4 in trimester 2) and severe (haemoglobin <7.0 g/dL) anaemia categories.

^c^Trimester of pregnancy defined as first (0–12 weeks), second (13–27 weeks) and third (28 weeks onwards).

^d^Levene’s test was significant. *T*-test results were interpreted based on equal variance not assumed (Welch two-sample *t*-test).

^e^Missing values: pregnancy trimester Hb measured (HF: *n* = 12, ULF: *n* = 20); gestational age at Hb measurement (HF: *n* = 12, ULF: *n* = 20).

*P is significant at <0.05, ** *P* is significant at <0.01, ***P is significant at <0.001.

**Table 2:** Maternal and infant sample characteristics according to antenatal maternal anaemia status across HF (3 T) and ULF (64 mT) subsamples

	HF subsample (*n* = 131)^a^			ULF Subsample (*n* = 205)^a^		
Variable^a^	Maternal anaemia^b^(*n* = 37)	No maternal anaemia^b^ (*n* = 94)	*P*	Maternal anaemia^b^ (*n* = 61)	No maternal anaemia^b^ (*n* = 144)	*P*
**Maternal characteristics**						
Monthly household income (ZAR)^c,d^						
< 1000	8 (21.62)	16 (17.02)	0.031*	12 (19.67)	24 (16.67)	0.022*
1000–5000	24 (64.86)	36 (38.30)		35 (57.38)	54 (37.50)	
5000–10 000	5 (13.51)	24 (25.53)		11 (18.03)	38 (26.39)	
> 10 000	0 (0.00)	8 (8.51)		0 (0.00)	10 (6.94)	
Education^c^						
Primary	1 (2.70)	3 (3.19)	0.405	1 (1.64)	2 (1.39)	
Some secondary	21 (56.76)	40 (42.55)		34 (55.74)	59 (40.97)	0.362
Completed secondary	12 (32.43)	30 (31.91)		20 (32.79)	60 (41.67)	
Some tertiary	2 (5.41)	11 (11.70)		4 (6.56)	13 (9.03)	
Completed tertiary	1 (2.70)	10 (10.64)		2 (3.28)	10 (6.94)	
Employed^d^	12 (32.43)	32 (34.04)	0.992	21 (34.43)	50 (34.72)	1
Age at enrolment (years)	29.75 (6.18) [20.70–40.50]	29.80 (5.42) [19.00–41.70]	0.965	29.18 (5.58) [18.50–40.30]	28.87 (5.75) [18.00–41.70]	0.723
Smoking during pregnancy^c^	1 (2.70)	3 (3.19)	1	4 (6.56)	3 (2.08)	0.200
Alcohol during pregnancy^c^	3 (8.12)	3 (3.19)	0.350	4 (6.56)	7 (4.86)	0.736
Depression during pregnancy	4 (10.81)	18 (19.15)	0.374	9 (14.75)	25 (17.36)	0.800
HIV infection during pregnancy	18 (48.65)	28 (29.79)	0.067	30 (49.18)	39 (27.08)	0.004**
**Infant characteristics**						
Sex (male)	17 (45.95)	48 (51.06)	0.739	24 (39.34)	74 (51.39)	0.154
Gestational age at birth (weeks)^d^	39.00 (2.08) [33.00–42.00]	39.30 (1.72) [32.00–42.00]	0.419	38.88 (2.02) [32.00–42.00]	39.23 (1.80) [30.00–42.00]	0.233
Child age at scan (months; *n* = 341)^e^	12.33 (6.78) [2.66–24.84]	11.77 (6.23) [2.56–24.11]	0.590	10.64 (5.43) [2.66–25.49]	11.13 (5.38) [2.69–25.07]	0.446
HIV infection^c,d^	0 (0.00)	0 (0.00)	N/A	1 (1.64)	0 (0.00)	0.436
Birth weight (g)^d,f^	3121.19 (486.63) [1975.00–4360.00]	3239.19 (513.51) [1810.00–4750.00]	0.233	3079.75 (471.40) [1975.00–4360.00]	3191.30 (511.11) [1180.00–4750.00]	0.146
Birth length (cm)^d,f^	49.61 (3.83) [41.00–57.00]	50.26 (3.12) [43.00–57.00]	0.296	49.34 (3.44) [41.00–57.00]	50.08 (3.54) [33.00–57.00]	0.177
Birth head circumference (cm)^d,f^	34.54 (1.69) [30.50–38.00]	34.82 (1.80) [31.00–40.00]	0.417	34.38 (1.60) [30.50–38.00]	34.55 (1.81) [28.00–40.00]	0.518

Abbreviations: HF, high-field; ULF, ultra-low-field; T, tesla; mT, millitesla; ZAR, South African Rand; HIV, human immunodeficiency virus; COVID-19, Coronavirus Disease 2019; g, grams; cm, centimetres.

Group differences based on antenatal maternal anaemia status were calculated using unpaired *t*-tests for continuous data and chi-squared tests or Fisher’s exact tests for categorical data.

^a^The total and group subsamples represent the number of unique subjects, excluding repeated measures data for children with multiple scans to avoid duplication of observations for infants with multiple scans. Values for continuous variables are presented as mean (standard deviation) [range]. Values for categorical variables are presented as count (%).

^b^Maternal anaemia during pregnancy was classified according to the WHO threshold of <11 g/dL for trimesters 1 and 3 and <10.5 g/dL for trimester 2. Severity categorizations were made indicating mild (haemoglobin 10.0–10.9 g/dL in trimesters 1 and 3; 9.5–10.4 in trimester 2), moderate (haemoglobin 7.0–9.9 g/dL in trimester 1; 7–9.4 in trimester 2) and severe (haemoglobin <7.0 g/dL) anaemia categories.

^c^Fisher’s exact test result interpreted due to one or more cells having an expected count of less than 5.

^d^Missing values: monthly household income (HF: *n* = 10, ULF: *n* = 21), maternal employment (HF: *n* = 1, ULF: *n* = 1), infant HIV infection *(HF: n* = 89, ULF: *n* = 143), child gestational age at birth (HF: *n* = 12 ULF: *n* = 20), infant birth weight (HF: *n* = 1, ULF: *n* = 2), infant birth length (HF: *n* = 2, ULF: *n* = 5), infant birth head circumference (HF: *n* = 2, ULF: *n* = 4).

^e^Mean child age at scan was calculated for the full subsample of scans (*n* = 341) including repeated measures, given that each child may have had multiple scans at different ages from different study visits. The group summary statistics for child age at scan represent the mean, standard deviation and range for each independent observation.

^f^The birth anthropometry was conducted by trained labour staff in the ward. Infant length was measured in centimetres to the nearest completed 0.5 cm, and weight was measured in kilograms.

*P is significant at <0.05, ** *P* is significant at <0.01, ***P is significant at <0.001.

In the ULF infant neuroimaging subsample (Table 1), 205 mothers [mean (SD) age at enrolment: 28.96 (5.69) years, age range of 18.00–41.70 years] had antenatal maternal haemoglobin data available. Minimum haemoglobin measurements were mostly acquired in the second [37.56% (77/205)] and third trimester of pregnancy (32.20% [66/205]), at a median (IQR) of 22 (13–30) weeks. Of these mothers, 29.76% (61/205) were classified as anaemic during pregnancy, with 67.21% (41/61) of these cases being mild, 31.15% (19/61) moderate and 1.64% (1/61) severe. With repeated measures available for 50.73% (104/205) of the subsample included (Fig. 2), a total of 341 infant scans [average age at time of scan: 10.98 (5.39) months, age range of 2.66–25.49 months] with corresponding maternal anaemia data were available. There were no differences in maternal characteristics or child characteristics between groups based on antenatal maternal anaemia status (*n* = 205; Table 2), with the exception of household income (*P* = 0.022) and antenatal maternal HIV (*P* = 0.004). Mothers with anaemia in pregnancy were found to have lower household income and a higher prevalence of HIV infection (49.18% versus 27.08%) than mothers without anaemia in pregnancy.

#### Exploratory analyses: antenatal maternal anaemia status

**Figure 3:**
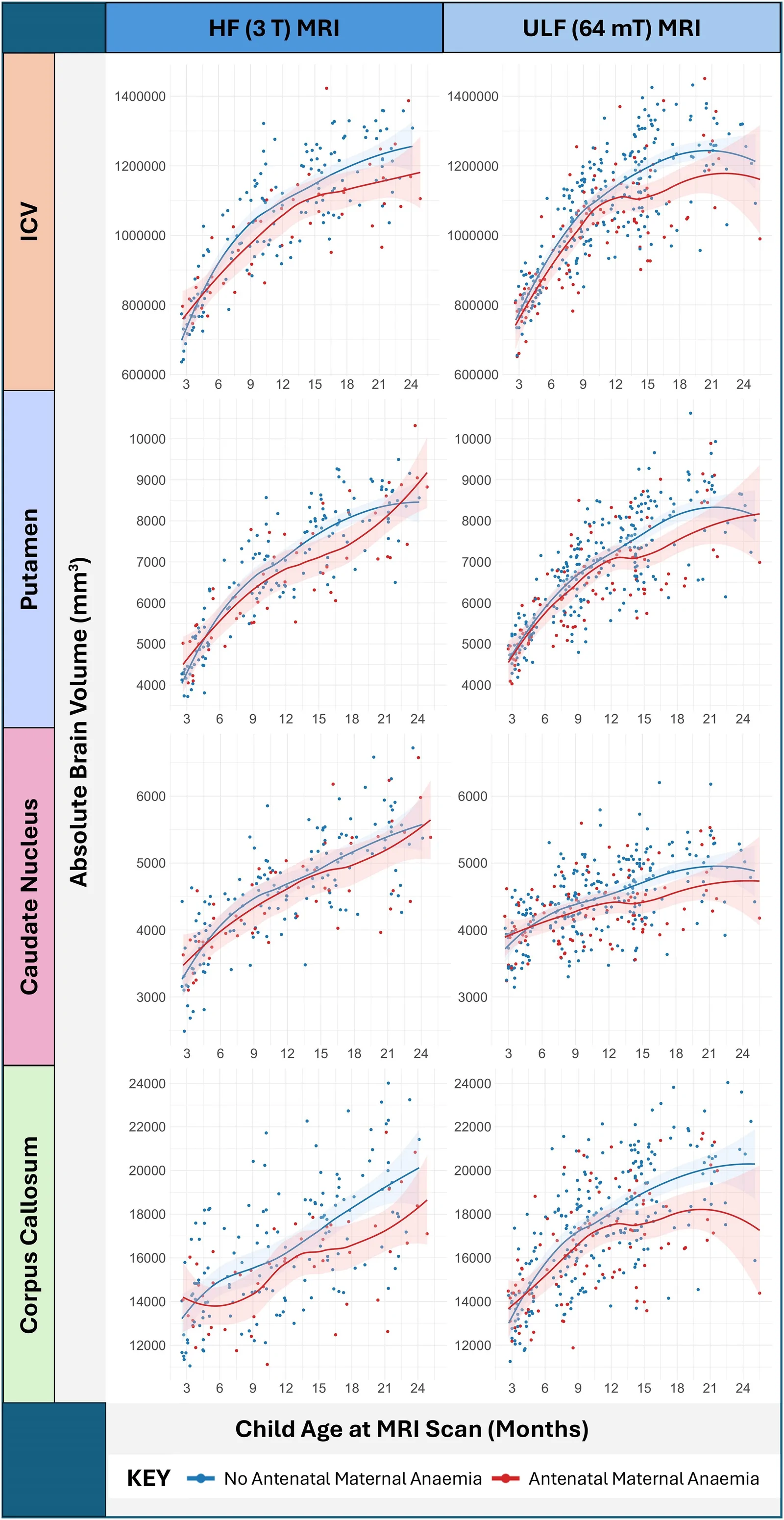
**HF (3 T; *n* = 195; left) and ULF (64 mT; *n* = 341; right) plots of absolute total and regional infant brain volumes by age and antenatal maternal anaemia status.** Each datapoint reflects an infant scan acquired at HF (left) or ULF (right) in repeated measures subsamples with 95% confidence intervals. The key demonstrates whether that infant was born to a mother with anaemia during pregnancy or a mother without anaemia during pregnancy.

In the full repeated measures subsamples of infants with HF (*n* = 195) and ULF (*n* = 341) neuroimaging data and corresponding antenatal maternal haemoglobin data, absolute brain volumes for ICV and ROIs were plotted against age (Fig. 3). LOESS curves were used to observe general trends in the developmental patterns of growth from 3 to 24 months. Overall, for both HF and ULF subsamples, trajectories for ICV and the basal ganglia structures (caudate nucleus and putamen) were characterized by rapid growth in early life that plateaued after 12 months of age. In contrast, the corpus callosum demonstrated a linear growth trajectory with age for the HF subsample (steady volume increase with age) and a non-linear growth trajectory for the ULF subsample (rapid growth followed by plateauing). Overall, the 95% confidence interval ribbons (Fig. 3) demonstrated some fluctuations with increased error at the tail end of the curves, likely reflecting less available data collected at older ages.

This general trajectory for ICV and absolute basal ganglia volumes was apparent in the HF subsample for both groups, with consistently lower volumes in children whose mothers were anaemic in pregnancy (Fig. 3). However, in the larger ULF subsample, a more prominent difference between groups was observed at 12 months, with lower ICV and basal ganglia volumes in infants of anaemic versus non-anaemic mothers thereafter. In contrast, the corpus callosum volume increased at a slower rate in infants whose mothers were anaemic during pregnancy than in infants whose mothers were not anaemic across both HF and ULF subsamples. Therefore, while corpus callosum volumes were similar across groups at 3 months of age, the observed differences became increasingly more prominent with age (Fig. 3).

#### Modelling: antenatal maternal anaemia status

The qualitative trends observed in exploratory analyses informed the use of age in the models, with absolute age being used for brain regions with linear growth trajectories and the logarithmic transformation of age being used for brain regions with non-linear growth trajectories. LMEs were established for the quantitative statistical assessment of observed trends in repeated measures data across groups by antenatal maternal anaemia status. Following on from the exploratory plots (Fig. 3), zero-order correlation matrices were compiled to preliminarily assess relationships between variables of interest for both HF (*n* = 195) and ULF (*n* = 341) datasets. Thereafter, LME models were fitted to assess the associations between antenatal maternal anaemia status and absolute regional brain volumes, with a random intercept to account for repeated measures. Based on the visually inferred non-linear relationship between child age and absolute ICV, putamen and caudate nucleus volumes across HF and ULF subsamples (Fig. 3), all LMEs for these regions were run with the logarithmic transformation of age. This data-driven approach was also applied for the corpus callosum, using absolute age for the HF subsample where a linear relationship was observed and the logarithmic transformation of age for the ULF subsample where a non-linear relationship was observed. Given that child age and child sex were correlated with all brain regions of interest and considering their known effect on brain structure,^42^ they were included as covariates in both HF and ULF models. Only main effects were tested for the HF subsample, while interactions between antenatal maternal anaemia status and age were explored for the larger ULF subsample. All models were conducted separately for each brain ROI.

Additional potentially confounding variables with observed differences in frequency between maternal anaemia groups (Table 2) were also considered for inclusion in the models based on zero-order correlations (Supplementary Material; Table S1). In the HF repeated measures subsample (*n* = 195), household income was lower in anaemic mothers (Table 2) but was not independently associated with total or regional brain volumes. In the ULF repeated measures subsample (*n* = 341), lower household income and maternal HIV (Table 2) were significantly correlated with each other and with antenatal maternal anaemia status. This suggests that women living with HIV in lower-income households may be at increased risk of being anaemic. However, neither was independently associated with total or regional brain volumes of interest. Therefore, to avoid multicollinearity, these covariates were excluded from HF and ULF models. Given that maternal HIV has been associated with altered child brain structure in previous research, we did consider its potentially confounding role in sensitivity analyses.

##### ICV

While no association between antenatal maternal anaemia and ICV was observed in the HF model (Table 3; *n* = 195; β = −0.15, *P* = 0.111), a significant main effect did emerge when assessed in a bigger subsample at ULF (Table 4; *n* = 341; β = −0.24, *P* = 0.004). Based on the final model assessing only main effects in the ULF subsample, the analysis of EMMs indicated that infants born to mothers with anaemia (EMM = 1 030 186.95 mm^3^, SE = 11 643.87 mm^3^) had 3.77% smaller ICV than infants born to mothers without (EMM = 1 070 599.18 mm^3^, SE = 7525.96 mm^3^). No interaction was observed between antenatal maternal anaemia and child age at ULF scan (β = −0.04, *P* = 0.311). However, the divergence between group ICV volume trajectories appeared more apparent at 12 months of age, suggesting that the effect of antenatal maternal anaemia on ICV may be more easily detectable in the second year of life (Fig. 3).

**Table 3:** Estimated marginal means and adjusted volume differences for final models in the HF (3 T) repeated measures subsample (*n* = 195)

Brain region	EMM volume (mm^3^)^a,b^				*P*
	Maternal anaemia (*n* = 51) EMM (SE)	No maternal anaemia (*n* = 144) EMM (SE)	Adjusted mean difference (95% CI)	Adjusted mean difference (%)	
ICV	1 015 568.33 (14 274.92)	1 042 549.15 (8899.98)	−26 981.82 (−60 277.08–6315.45)	−2.59	0.111
Putamen	6643.95 (111.70)	6770.92 (69.61)	−127.04 (−387.56–133.48)	−1.88	0.336
Caudate nucleus	4540.91 (84.30)	4577.25 (52.46)	−36.32 (−232.87–160.18)	−0.79	0.715
Corpus callosum	15 594.77 (351.67)	16 449.43 (218.51)	−854.66 (−1674.16–35.15)	−5.20	0.041*

Abbreviations: HF, high-field; T, tesla; EMM, estimated marginal mean; SE, standard error; CI, confidence interval; ICV, intracranial volume.

^a^LME models were fitted for HF data with antenatal maternal anaemia status, child age at scan, child sex and a random intercept to account for repeated measures. Absolute child age in months was entered for brain regions with linear growth (corpus callosum), and the logarithmic transformation of age was entered for brain regions with non-linear growth (ICV, putamen, caudate nucleus). No interactions were examined for HF model fitting due to limited power and sparse sample distribution across age groups.

^b^EMMs are based on the regression line for fully adjusted models. These means are estimated based on the fit of the data, taking into account the role of other covariates.

*P is significant at <0.05, ** *P* is significant at <0.01, ***P is significant at <0.001.

**Table 4:** Estimated marginal means and adjusted volume differences for final models in the ULF (64 mT) repeated measures subsample (*n* = 341)

Brain region	Age group^c^	EMM volume (mm^3^)^a,b^				*P*
		Maternal anaemia (*n* = 101) EMM (SE)	No maternal anaemia (*n* = 240) EMM (SE)	Adjusted mean difference (95% CI)	Adjusted mean difference (%)	
ICV^d^	N/A	1 030 186.95 (11 643.87)	1 070 599.18 (7525.96)	− 40 413.23 (−67 783.01–13 043.46)	−3.77	0.004**
Putamen^d^	N/A	6614.21 (91.16)	6841.27 (59.43)	− 227.06 (−443.21–−10.92)	−3.32	0.04*
Caudate nucleus^e^	3	3772.39 (75.76)	3725.09 (52.19)	− 47.31 (−138.69–233.30)	1.27	0.617
	6	4113.12 (59.27)	4153.31 (39.01)	− 40.19 (−180.12–99.75)	−0.97	0.572
	12	4453.85 (57.71)	4581.52 (36.85)	−127.67 (−262.75–7.41)	−2.79	0.064
	18	4653.16 (66.22)	4832.02 (41.75)	−178.85 (−333.01–−24.69)	−3.70	0.023*
	24	4794.58 (75.19)	5009.74 (47.24)	−215.16 (−389.92–−40.21)	−4.29	0.016*
Corpus callosum^e^	3	13 382.54 (339.57)	13 183.07 (225.28)	199.47 (−602.74–1001.69)	1.51	0.625
	6	15 380.42 (252.94)	15 679.28 (166.64)	−298.86 (−896.20–298.47)	−1.91	0.325
	12	17 378.29 (245.89)	18 175.49 (156.91)	−797.20 (−1372.69–−221.71)	−4.39	0.007**
	18	18 546.97 (283.85)	19 635.68 (178.82)	−1088.71 (−1749.38–−428.03)	−5.54	0.001**
	24	19 376.17 (323.67)	20 671.70 (203.26)	−1295.54 (−2047.70–−543.36)	−6.27	0.001**

Abbreviations: ULF, ultra-low-field; mT, millitesla; EMM, estimated marginal mean; SE, standard error; CI, confidence interval; ICV, intracranial volume.

^a^LME models were fitted for ULF data with antenatal maternal anaemia status, child age at scan, child sex and a random intercept to account for repeated measures. The logarithmic transformation of age was entered for all brain regions with non-linear growth (ICV, putamen, caudate nucleus, corpus callosum). Interactions between antenatal maternal anaemia status and infant age at scan were also investigated. For brain regions with no significant interaction (ICV and putamen), the model was re-run without the interaction for the assessment of main effects.

^b^EMMs are based on the regression line for fully adjusted models. These means are estimated based on the fit of the data, taking into account the role of other covariates.

^c^EMMs were reported for overall groups (including all timepoints between 3 and 24 months) in models with no significant interaction between antenatal maternal anaemia status and age. For models with a significant interaction between antenatal maternal anaemia status and age, the EMMs and adjusted differences were reported by specified age groups between 3 and 24 months.

^d^Model with no significant interaction. Only main effects were interpreted.

^e^Model with a significant interaction. EMMs and multiple pairwise comparisons were reported by timepoint.

*P is significant at <0.05, ** *P* is significant at <0.01, ***P is significant at <0.001.

Child age (HF: β = 0.80, *P* < 0.001; ULF: β = 0.80, *P* < 0.001) and child sex (HF: β = 0.40, *P* < 0.001; ULF: β = 0.42, *P* < 0.001) were also significant predictors in both HF and ULF models, with larger ICV observed in boys and with increasing age.

##### Corpus callosum

In the primary LME model for the HF subsample (Table 3; *n* = 195), antenatal maternal anaemia was associated with corpus callosum volumes (β = −0.30, *P* = 0.041). Analysis of EMMs indicated that infants born to mothers with anaemia (EMM = 15 594.77 mm^3^, SE = 351.67 mm^3^) had 5.20% smaller volumes of the corpus callosum than infants born to mothers without (EMM = 16 449.43 mm^3^, SE = 218.51 mm^3^). Building on this, an interaction between age and antenatal maternal anaemia status was detected with ULF (Table 4; *n* = 341; β = −0.15, *P* = 0.007), indicating that the impact of antenatal maternal anaemia status on corpus callosum volumes may vary in magnitude with age. Analysis of *post hoc* EMMs revealed that group differences in corpus callosum volumes only became evident at 12 months of age (*P* = 0.007). In this age group, children born to mothers with anaemia in pregnancy (EMM: 17 378.29 mm^3^, SE = 245.89 mm^3^) had 4.39% smaller corpus callosum volumes than children born to mothers without (EMM = 18 175.49 mm^3^, SE = 156.91 mm^3^). However, by 24 months of age (*P* = 0.001), children born to mothers with anaemia in pregnancy (EMM = 19 376.17 mm^3^, SE = 323.67 mm^3^) had 6.27% smaller corpus callosum volumes than children born to mothers without (EMM = 20 671.70 mm^3^, SE = 203.26 mm^3^). Overall, the difference in corpus callosum volumes between groups became more prominent with age in the first 2 years of life (Fig. 3).

Child sex (HF: β = 0.34, *P* = 0.011; ULF: β = 0.47, *P* < 0.001) and child age (HF: β = 0.56, *P* < 0.001; ULF: β = 0.75, *P* < 0.001) were also significant predictors in the model, suggesting that increasing age and being biologically male were associated with larger corpus callosum volumes in infants between 3 and 24 months of age, both expected findings.

##### Caudate nucleus and putamen

In the primary model for the HF subsample (Table 3; *n* = 195), antenatal maternal anaemia was not associated with caudate nucleus (β = −0.04, *P* = 0.715) or putamen volumes (β = −0.08, *P* = 0.336). However, based on the final model assessing only main effects in the ULF subsample (Table 4; *n* = 341), antenatal maternal anaemia was negatively associated with putamen volumes (β = −0.18, *P* = 0.040). Analysis of EMMs in the ULF subsample indicated that infants born to mothers with anaemia (EMM = 6614.21 mm^3^, SE = 91.96 mm^3^) had 3.32% smaller putamen volumes than infants born to non-anaemic mothers (EMM = 6841.27 mm^3^, SE = 59.43 mm^3^). No interaction was observed between antenatal maternal anaemia and child age for the putamen at ULF (β = −0.03, *P* = 0.519). In contrast, the caudate nucleus was associated with a significant interaction between age and antenatal maternal anaemia status (β = −0.13, *P* = 0.038). This suggests that the impact of antenatal maternal anaemia status on caudate nucleus volumes may vary in magnitude with age, with between-group differences emerging after 12 months of age (Fig. 3). Analysis of *post hoc* EMMs revealed that group differences in caudate nucleus volumes only became statistically detectable at 18 months of age (*P* = 0.023). In this age group, children born to mothers with anaemia in pregnancy (EMM: 4653.16 mm^3^, SE = 66.22 mm^3^) had 3.70% smaller caudate nucleus volumes than children born to mothers without (EMM = 4832.02 mm^3^, SE = 41.75 mm^3^). By 24 months of age (*P* = 0.016), children born to mothers with anaemia in pregnancy (EMM = 4794.58 mm^3^, SE = 75.19 mm^3^) had 4.29% smaller caudate nucleus volumes than children born to mothers without (EMM = 5009.74 mm^3^, SE = 47.24 mm^3^).

As expected, child age (HF: β = 0.86, *P* < 0.001; ULF: β = 0.83, *P* < 0.001) and child sex (HF: β = 0.29, *P* < 0.001; ULF: β = 0.35, *P* < 0.001) were positively associated with putamen volumes in both HF and ULF models. This relationship for child age (HF: β = 0.72, *P* < 0.001; ULF: β = 0.63, *P* < 0.001) and sex (HF: β = 0.30, *P* = 0.007; ULF: β = 0.46, *P* < 0.001) was also observed for the caudate nucleus. As observed for ICV and corpus callosum volumes, this suggests larger basal ganglia volumes in boys and with increasing age.

#### Sensitivity analyses

**Figure 4:**
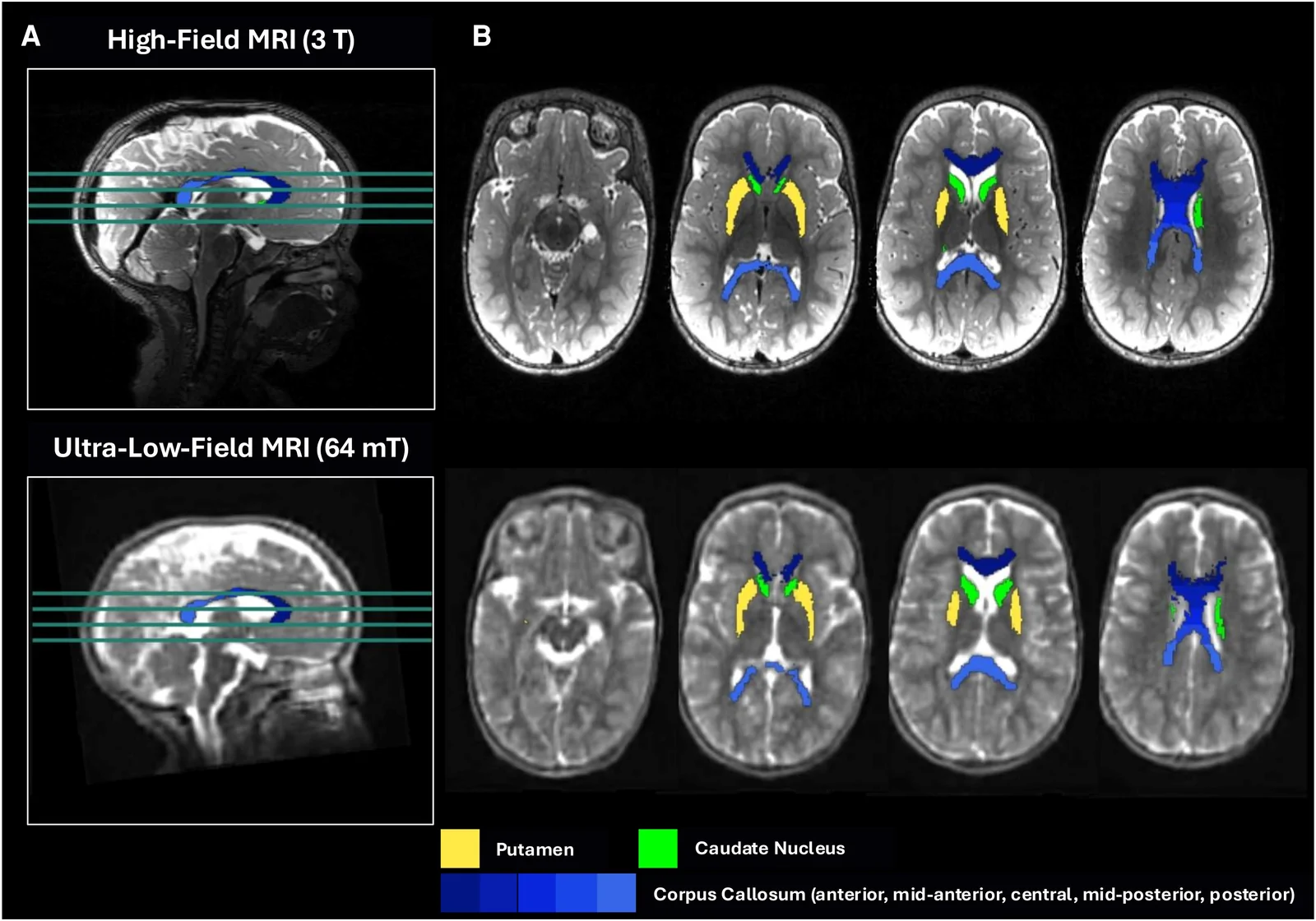
**Segmentation overlay of brain regions associated with antenatal maternal anaemia in an infant at 12 months of age scanned using HF (3 T) and ULF (64 mT) MRI.** The brain images were created using MRIcroGL software (Version 15.0.1). (**A**) Sagittal view of the brain demonstrating slices from inferior (bottom) to superior (top). (**B**) Corresponding axial slices with segmentation overlay for brain regions of interest from inferior (left) to superior (right).

Overall, there was good segmentation consistency across field strengths, and antenatal maternal anaemia was associated with smaller total and regional brain volumes in infants between 3 and 24 months of age in both HF and ULF subsamples (Fig. 4). However, there was more statistical power to detect significant findings in the larger ULF dataset. While maternal HIV was not correlated with regional brain volumes (Supplementary Material; Table S1), it has previously been associated with altered child brain structure.^44^ Therefore, it was included as a binary variable in sensitivity analyses to ensure no confounding effects in the ULF subsample where group differences were observed. In sensitivity analyses comparing results with and without the inclusion of maternal HIV status across models, the effect of antenatal maternal anaemia on child brain structure remained robust and maternal HIV did not play a significant role in this subsample.

### Secondary analysis: postnatal child anaemia status | ULF MRI

#### Sample characteristics: postnatal child anaemia status

In the ULF neuroimaging subsample (Supplementary Material; Table S2), 214 children had haemoglobin data from at least one study visit between 3 and 24 months. Of these children, 48.13% (103/214) were anaemic based on the lowest haemoglobin measurement recorded during this period. On average, the minimum postnatal haemoglobin measurements from across scans for each infant (*n* = 214) were acquired around the 6-month visit [mean (SD) = 7.11 (4.22) months, range = 2.01–18.67 months]. With repeated measures, 352 children had scans [average age at time of scan: 11.70 (5.54) months, age range of 2.66–25.49 months] with corresponding data on anaemia status based on the lowest haemoglobin measure between birth and the time of scan. Risk factors for child anaemia were identified (Supplementary Material; Table S2) as younger gestational age at birth (*P* = 0.034) and lower child anthropometry outcomes (birth length, *P* = 0.027). There were no other differences in maternal characteristics or infant characteristics between groups based on child anaemia status.

#### Modelling: postnatal child anaemia status

**Figure 5:**
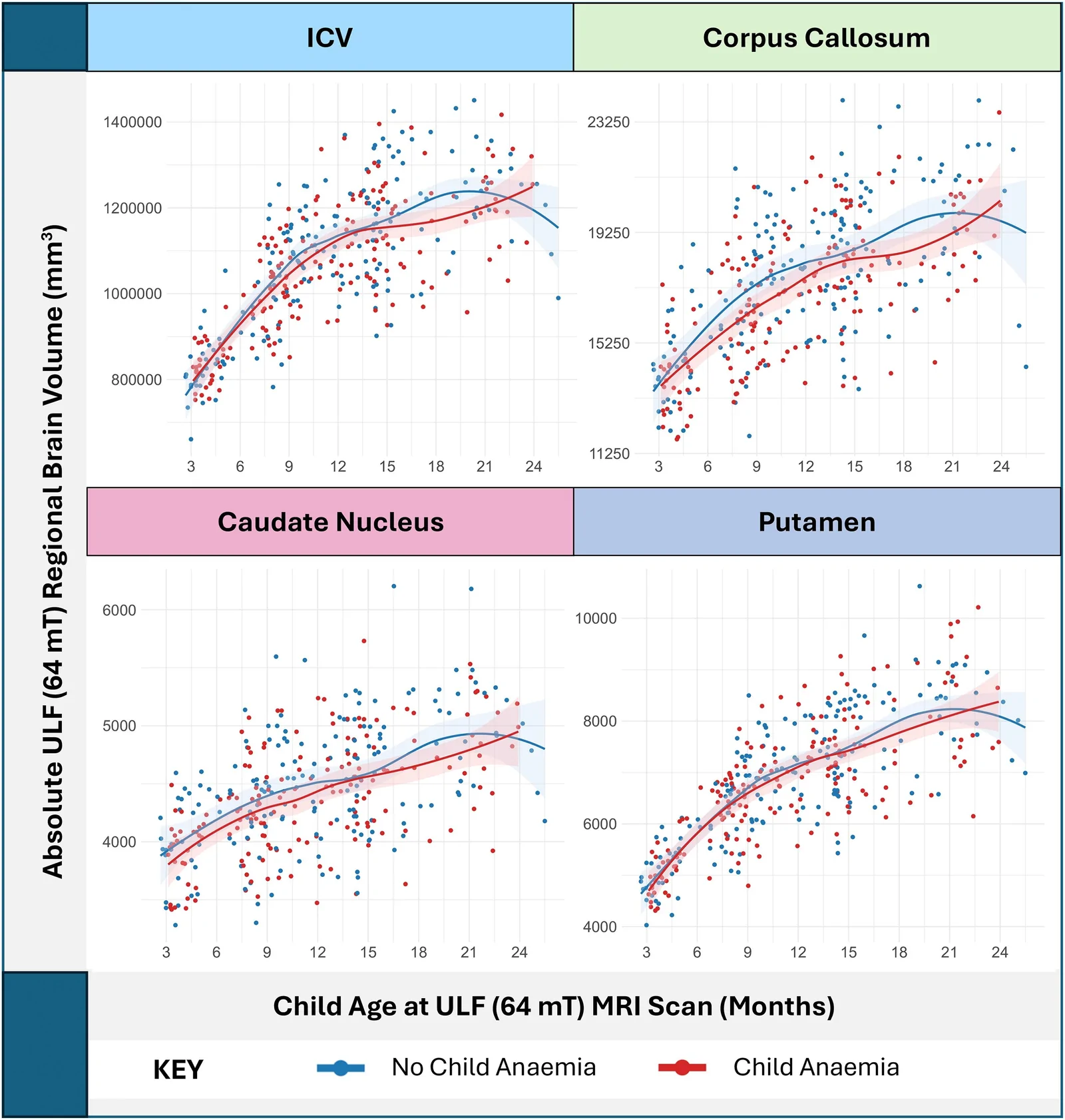
**ULF (64 mT) total brain volume and regional infant brain volumes by age and child anaemia status (*n* = 352).** Each datapoint reflects an infant scan acquired at ULF in a repeated measures subsample with 95% confidence intervals. The key demonstrates whether that infant was anaemic or not anaemic based on the minimum haemoglobin measure observed between birth and the time of scan.

As an exploratory step, absolute brain volumes for ICV and ROIs were plotted against age with separate LOESS curves for children with and without anaemia in the repeated measures subsample (*n* = 352; Fig. 5). No prominent group differences were observed on visual inspection. This was confirmed by LME models (with logarithmic transformation of age, child sex and child anaemia status as predictors of interest) demonstrating no independent relationship between child anaemia and child brain volumes for ICV (β = −0.03, *P* = 0.698) or the corpus callosum (β = −0.14, *P* = 0.123), caudate nucleus (β = −0.13, *P* = 0.199) and putamen (β = −0.02, *P* = 0.744). Following on from this, the final antenatal maternal anaemia models (Primary Analysis) were replicated with the addition of child anaemia status as a covariate to assess the relative roles of antenatal versus postnatal anaemia exposure (*n* = 289). While the effects of antenatal maternal anaemia status remained similar, postnatal child anaemia was not significantly associated with ICV (β = −0.04, *P* = 0.572), corpus callosum (β = −0.15, *P* = 0.129), caudate nucleus (β = −0.21, *P* = 0.059) or putamen (β = −0.01, *P* = 0.942) volumes.

### Tertiary analysis: iron deficiency status | ULF MRI

Given that antenatal maternal anaemia (but not postnatal child anaemia) was significantly associated with ICV and regional child brain volumes, the role of iron deficiency was only investigated in mothers. This was based on the motivation for exploring the role of iron deficiency as a neurobiological mechanism underlying associations between anaemia and child brain structure.

#### Sample characteristics: antenatal maternal iron deficiency status

In the ULF infant neuroimaging subsample (Supplementary Materials; Table S3), 73 mothers [mean (SD) age at enrolment: 28.80 (5.78) years, age range of 19.00–40.3 years] had adjusted serum ferritin data from pregnancy available. Minimum serum ferritin measures were predominantly acquired in the third trimester of pregnancy [38.36% (28/73)], at a median (IQR) of 33 (30–35) weeks of gestation. Of these mothers, 39.73% (29/73) were classified as iron deficient during pregnancy after adjustment for inflammation. With repeated measures (*n* = 128), infants [average age at time of scan: 10.56 (5.32) months, age range of 2.69–25.07 months] born to mothers with iron deficiency in pregnancy had similar maternal and infant characteristics to infants born to mothers without iron deficiency in pregnancy.

#### Modelling: antenatal maternal iron deficiency status

**Figure 6:**
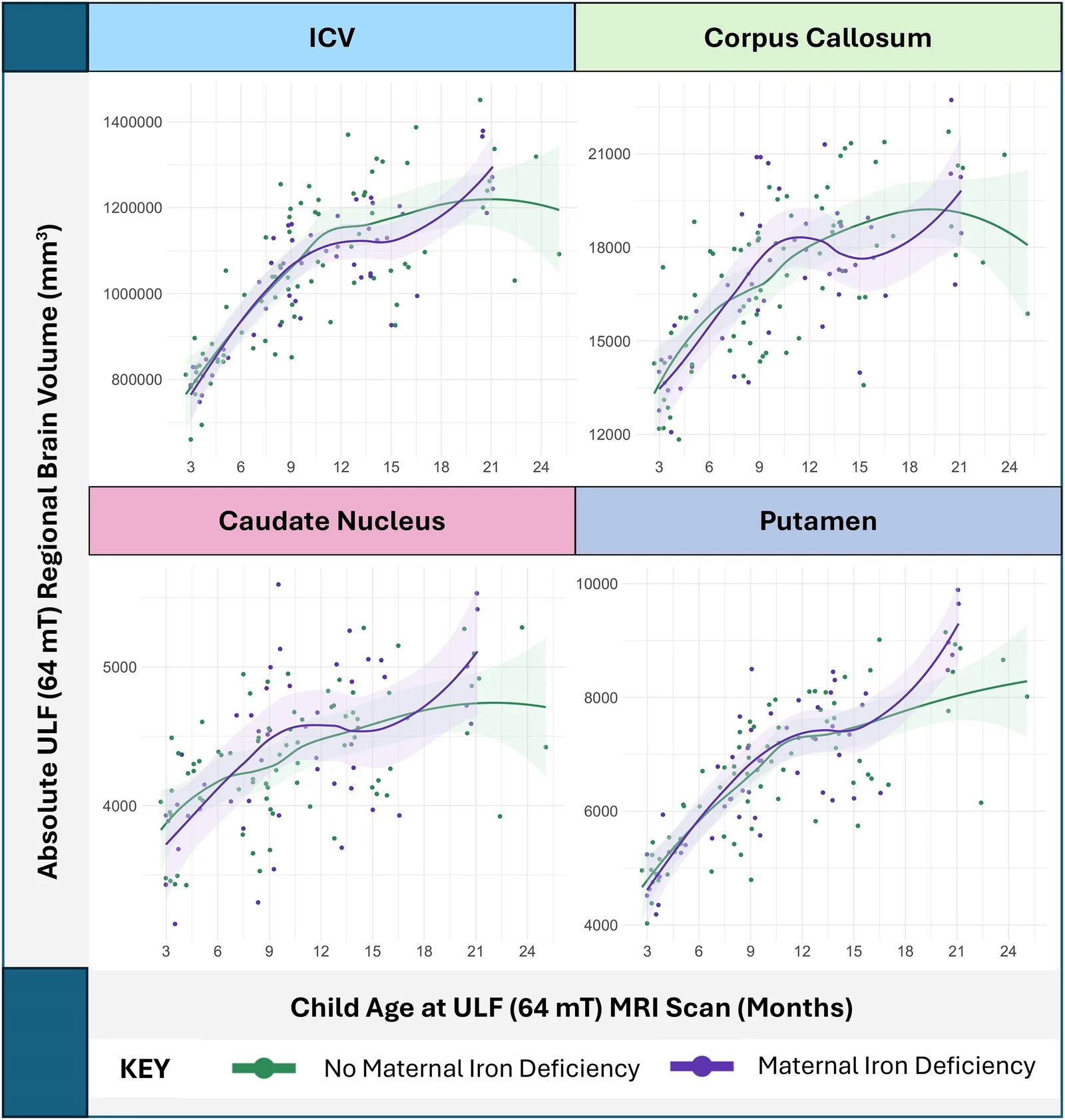
**ULF (64 mT) total brain volume and regional infant brain volumes by age and antenatal maternal iron deficiency status (*n* = 128).** Each datapoint reflects an infant scan acquired at ULF in a repeated measures subsample with 95% confidence intervals. The key demonstrates whether that infant was born to a mother with iron deficiency during pregnancy or a mother without iron deficiency during pregnancy.

As an exploratory step, absolute brain volumes for ICV and ROIs were plotted against age using LOESS curves (*n* = 128; Fig. 6). Overall, no consistent group differences based on antenatal maternal iron deficiency status were observed. However, the 95% confidence interval ribbons demonstrated significant error margins, particularly in older children, likely due to the small sample size and data sparsity in this age group. Based on LME models (log of age, child sex, antenatal maternal iron deficiency as predictors of interest) assessing main effects, antenatal maternal iron deficiency was not associated with child brain volumes for ICV (β = −0.13, *P* = 0.317) or the corpus callosum (β = −0.05, *P* = 0.791), caudate nucleus (β = 0.06, *P* = 0.767) and putamen (β = 0.05, *P* = 0.730).

## Discussion

With the emerging recognition of the important role of antenatal maternal anaemia in altered neurodevelopment, there is a growing need to fully understand its impact across childhood. This neuroimaging work builds on previous South African research on toddlers^8^ and children,^22^ providing novel insight into the effects of anaemia in early infancy on brain structural growth. As the first study to explore these relationships in children under the age of 2 years, the results also suggest the age at which the effects of antenatal maternal anaemia may be most clearly detectable in the volumes of key subcortical structures. Overall, maternal anaemia in pregnancy, even when mild, was associated with smaller total (ICV) and regional (corpus callosum, caudate nucleus and putamen) infant brain volumes (Fig. 4), with group differences becoming detectable at ∼12 months of age. These effects were observed using both HF and ULF scanners, informing the feasibility of ULF MRI for ongoing research on anaemia in babies and young children from low-resource settings. Given the longitudinal nature of the cohort, these results inform current knowledge of regional brain volume trajectories in an LMIC context. With most brain growth curves being based on data from high-income countries,^45^ this is key for the valid interpretation of neuroimaging data on prevalent risk factors for child brain development across Africa.

Based on paediatric neuroimaging data from 3 to 24 months acquired for the ULF subsample, non-linear growth trajectories were observed using LOESS curves for ICV as well as the corpus callosum, caudate nucleus and putamen. This was characterized by rapid growth in early life with plateauing after 12 months of age. These patterns of structural brain development in South African babies from the Khula study are consistent with global brain charts across the lifespan^45^ and studies focusing on volumetric development in the first 2 years of childhood.^46-48^ Overall, growth trajectories were broadly comparable across field strengths for all brain regions, with the exception of the corpus callosum. In contrast to the non-linear corpus callosum growth trajectory described for the ULF subsample above, the corpus callosum appeared to follow a linear trajectory in the HF subsample. This apparent discrepancy may reflect differences in sample size, with the smaller HF subsample being less powered to fully capture the expected variability in brain volumes across the first 2 years of life. However, previous cross-validation work in this cohort has shown lower concordance between 3 T and 64 mT measurements for the corpus callosum,^24^ suggesting that this discrepancy may also partly reflect measurement error.

As hypothesized, maternal anaemia in pregnancy was associated with reduced total brain volume (ICV) and smaller absolute volumes of the corpus callosum, caudate nucleus and putamen. Given the similarities in overall brain growth trajectories and observed volumetric differences by maternal anaemic status across MRI field strengths, this study suggests that the ULF system may provide sufficient sensitivity for detecting the effects of anaemia on the infant brain. This finding is key in validating the use of ULF MRI for both observational and experimental studies on the effects of risk factors and outcomes of interventions in ongoing anaemia research across the UNITY (Ultra- Low-Field Neuroimaging In The Young) network.^23^ This represents an important advancement towards increasing neuroimaging accessibility in LMICs faced with the highest burden of disease.

In the ULF subsample, infants born to mothers with anaemia in pregnancy had ∼4% smaller total brain volumes and ∼3% smaller putamen volumes. These group differences became more prominent after 12 months, with a steeper deceleration in growth at this age. Even larger effects were demonstrated for the caudate nucleus and corpus callosum, with an apparent interaction between antenatal maternal anaemia status and age across the first 2 years of life. Maternal anaemia in pregnancy was associated with ∼4% smaller volumes of the caudate nucleus, with this effect becoming visually pronounced at 12 months (flatter plateauing of the growth curve in exposed infants) and statistically significant for the first time at 18 months of age. Similarly, infants born to mothers who were anaemic during pregnancy had ∼4% smaller corpus callosum volumes by 12 months and ∼6% smaller volumes by 24 months, with a slower rate of non-linear growth across the first 2 years of life in exposed infants. Overall, the impact of antenatal maternal anaemia differed in magnitude by age, with effects becoming statistically detectable as infants approached 1 year of age.

These results from the Khula cohort are unique as they are the first to reveal the impact of antenatal maternal anaemia on child brain structure as early as infancy, when postnatal factors are less likely to have played as much of a role. However, they are consistent with previously reported research on toddlers^8^ and children^22^ in a different South African cohort (DCHS), demonstrating remarkably comparable adjusted volume differences across age groups and studies for the corpus callosum (4% smaller at 1 year and 6% smaller at 2 years in the Khula cohort, 7–8% smaller at 2–3 and 6–7 years in the DCHS cohort), caudate nucleus (4% smaller at 1.5–2 years in the Khula cohort, 5–6% smaller at 2–3 and 6–7 years in the DCHS cohort) and putamen (3% smaller across first 2 years in the Khula cohort, 4% smaller at 2–3 years in the DCHS cohort). Taken together, these findings corroborate antenatal maternal anaemia as an important risk factor for altered child brain structure, with regionally consistent volumetric differences becoming statistically detectable at 12 months and persisting throughout the first 6–7 years of childhood. The replication of study results across these two representative cohorts also suggests that the demonstrated effects of anaemia on neurodevelopment may be generalizable across other communities in South Africa.

While this work reinforces the importance of pregnancy as the key period for optimized intervention, it also suggests the first year of life as a postnatal window during which structural brain differences in children exposed to maternal anaemia are not yet fully detectable. Based on previous research from the same cohort, the 12-month timepoint is also associated with an increased risk of postnatal iron deficiency as well as anaemia in infants born to anaemic mothers.^13^ It is possible that foetal iron loading^10,11^ and immuno-protective breastmilk with high iron bioavailability^49^ may serve as a buffer in the first year of life, reducing the risk of child iron deficiency anaemia and mitigating the effects of antenatal maternal anaemia on child brain structure during this period. This is relevant given its potential implications for the extension of targeted anaemia treatment strategies (e.g. iron supplementation) aimed at leveraging protective factors in the first 12 months of infancy. This may be particularly important for children at increased risk of anaemia themselves due to limited foetal iron loading in utero (antenatal maternal iron deficiency or preterm birth)^50^ and postnatal factors such as poor nutrition or infection in early life.^13^

However, similar to what was observed in toddlers^8^ and school-age children^22^ from the DCHS, child anaemia was not independently associated with infant brain volume differences compared to those who were not anaemic in this Khula study. Furthermore, child anaemia did not attenuate or change the overall effect of antenatal maternal anaemia on child brain volumes. While it is possible that child anaemia may still impact other aspects of brain development such as white matter maturation, volumetric outcomes seem to be largely driven by maternal anaemia in pregnancy. On this basis, the detection of group differences at 12 months is unlikely to be caused by an interaction between antenatal and postnatal factors. Instead, the neurodevelopmental effects of anaemia in pregnancy may have been present earlier than 12 months but masked by rapid overall brain growth and simultaneous asynchronous volume increases in other regions.^47,51^ With the plateauing and stabilization of ICV and regional brain volumes from 12 months onwards, pre-existing patterns may have simply become clearer for disentanglement and detection. Therefore, while the first year of life presents an opportunity for further investigation and caution, we emphasize the importance of timing, with the pregnancy period remaining critical for optimized outcomes.

Prevention and intervention strategies could be improved by targeting the underlying aetiologies of anaemia. Given that ∼50% of anaemic cases can be attributed to iron deficiency in this cohort,^13^ this was investigated as an independent risk factor for altered child brain structure. However, the results of this study found no group differences in total or regional infant brain volumes based on maternal iron deficiency status in pregnancy. In explaining this finding, it is noted that the analysis on iron deficiency status may have been limited by insufficient power due to having half the sample size used for the anaemia analysis. While there are methodological limitations to our interpretation of this finding, the results do suggest that an absolute iron deficiency in mothers may not be the key neurobiological driver of observed associations between anaemia and regional infant brain volumes. Additionally, HIV was more common in mothers with antenatal maternal anaemia, suggesting that ‘anaemia of chronic disease’ may be the most relevant mechanism in this cohort. In these cases, chronic inflammation associated with infectious disease could be contributing to a functional iron deficiency characterized by sufficient iron stores but limited iron bioavailability.

Given that serum ferritin is an acute phase reactant that becomes transiently elevated in high inflammatory states, this study adjusted serum ferritin concentrations based on acute and chronic inflammatory biomarkers. Although this was necessary for the accurate classification of iron deficiency status, it does not preclude the possibility of iron stores not being accessible for use due to increased sequestration in the case of inflammation. If the effect of anaemia on child brain structure is being driven by a functional iron deficiency, comparing iron deficiency status based on absolute (albeit adjusted) serum ferritin values may not be appropriate. Future studies should focus on inflammatory mechanisms less sensitive to inflammation, such as transferrin saturation (TSAT)^52-55^ and reticulocyte haemoglobin (Ret-He) concentration,^56^ to make comparisons based on iron bioavailability. Additionally, the inclusion of data on immune health in HIV (viral load and CD4 counts), alongside inflammatory biomarkers and iron metrics, may provide a more nuanced understanding of the biological relationship between IDA and HIV as overlapping risk factors for altered child brain development.

In conclusion, antenatal maternal anaemia emerged as an important factor associated with reduced total brain volume as well as smaller corpus callosum, caudate nucleus and putamen volumes. These findings were evident even with most cases of maternal anaemia being mild. This study is the first to demonstrate that the impact of maternal anaemia in pregnancy on child brain structure is evident as early as infancy, with effects becoming detectable on brain volumes at 12 months of age. Although child anaemia was not associated with smaller total or regional brain volumes, the first year of life may present as an extended window for further intervention. Mothers of low household income living with HIV were at greatest risk of anaemia in pregnancy, suggesting that food insecurity due to poor socio-economic status and inflammation associated with infectious disease may be important factors for consideration. Antenatal maternal iron deficiency was not associated with smaller total or regional infant brain volumes; however, this analysis was limited by a smaller sample. Future research should include additional iron metrics for the assessment of iron bioavailability status in mothers with high levels of inflammation due to infectious disease, and intervention strategies should focus on the treatment of anaemia in conjunction with appropriate infection management.

Alongside informing global recommendations for the timing and optimization of anaemia management in both clinical practice and policy, it is important that the research reaches the communities who stand to benefit the most from the implications. As a public engagement project, key study findings were used to inform the development of an educational video resource entitled ‘Nourishment and Neurodevelopment: The Importance of Nutrition for Your Child’s Brain Health’.^57^ The content was guided by reciprocal dialogue between researchers with expertise in child development, mothers from the study community and a dietician familiar with the common practices, practical considerations and challenges experienced in this context. The series is aimed at mothers and caregivers and is available in English as well as two South African languages [Afrikaans and isiXhosa] on the University of Cape Town’s website: Public Engagement for Neurodevelopment and Neuroimaging of The Young (PENNY). Lastly, given the demonstrated validity of ULF MRI for research on anaemia in infants and young children, this study corroborates its use as a valuable tool for ongoing research and trials in LMICs around the world.

## Supplementary Material

fcag318_Supplementary_Data

## Data Availability

The de-identified data that support the findings of this study are available from the authors upon reasonable request as per the Khula South Africa study guidelines. All software used in the development of Flywheel gears for the UNITY project has been shared as an open-source resource on GitHub (https://github.com/UNITY-Physics). This includes the MiniMORPH processing pipeline (https://github.com/UNITY-Physics/fw-minimorph) and the R script used for statistical analyses (https://github.com/JERingshaw/Khula-Anaemia-Imaging-Analysis.git)

## Appendix I

Khula South Africa Data Collection Team: Lauren Davel, Tracy Pan, Tembeka Mhlakwaphalwa, Bokang Methola, Khanyisa Nkubungu, Candice Knipe, Zamazimba Madi, Nwabisa Mlandu, Ringie Gulwa, Pamela Madikane.
