# Supplementary material for: Antenatal maternal anaemia and infant brain structure: high-field (3 T) and ultra-low-field (64 mT) MRI findings from South Africa
Source: Brain Commun. 2026 Sep 8;8(5):fcag318. doi: 10.1093/braincomms/fcag318 (PMC13553114; doi:10.1093/braincomms/fcag318)
Supplement: Supplementary file 1 [file fcag318_supplementary_data.pdf]

SUPPLEMENTARY MATERIAL

ANTENATAL MATERNAL ANAEMIA AND INFANT BRAIN STRUCTURE: HIGH-FIELD (3 T) AND ULTRA-LOW-FIELD (64 mT) MRI FINDINGS FROM SOUTH AFRICA

Table S1. Zero-order correlations between potential confounders, antenatal maternal anaemia status, and child brain volumes

Table S2. Maternal and infant characteristics for the ultra-low-field (ULF; 64 mT) subsample according to child anaemia status

Table S3. Maternal and infant characteristics for the ultra-low-field (ULF; 64 mT) subsample according to BRINDA-adjusted antenatal maternal iron deficiency status

Table S1. Zero-order correlations between potential confounders, antenatal maternal anaemia status, and child brain volumes

Correlation Matrix *r (p)*							
**Variable[a]**	**Household Income**	**Maternal HIV**	**Maternal Anaemia**	**ICV**	**Corpus Callosum**	**Caudate Nucleus**	**Putamen**
**HF (3 T) Subsample (n = 195)**							
**Household Income**	-	-0.278 (<0.001)***	-0.127 (0.087)	-0.099 (0.182)	-0.003 (0.968)	-0.03 (0.690)	-0.10 (0.176)
**Maternal HIV**		-	0.103 (0.151)	0.002 (0.983)	-0.026 (0.716)	-0.029 (0.683)	-0.008 (0.914)
**ULF (64 mT) Subsample (n = 341)**							
**Household Income**	-	-0.241 (<0.001)***	-0.189 (0.001)	0.055 (0.333)	0.019 (0.737)	0.105 (0.066)	0.058 (0.309)
**Maternal HIV**		-	0.131 (0.015)*	0.028 (0.605)	0.031 (0.566)	-0.029 (0.589)	-0.028 (0.610)

Abbreviations: HF, high-field; ULF, ultra-low-field; T, tesla; mT, millitesla; HIV, Human Immunodeficiency Virus; ICV, intracranial volume.

[a] Covariates with observed differences in frequency between mothers with anaemia and mothers without anaemia were considered as potential confounders. Their inclusion in models assessing associations between antenatal maternal anaemia status and regional child brain volumes was considered based on zero-order correlations.

**p* is significant at <0.05, ***p* is significant at <0.01, ****p* is significant at <0.001.

Table S2. Maternal and infant characteristics for the ultra-low-field (ULF; 64 mT) subsample according to child anaemia status

**Variable**[a]	**Total Subsample (*n* = 214)**[a]		
	**Child Anaemia**[b] (***n*** **= 103)**	**No Child Anaemia**[b] (***n*** **= 111)**	***p***
**Maternal Characteristics**			
Monthly household income (ZAR) [e,f]			
<1000	15 (14.56)	22 (19.82)	0.430
1000-5000	52 (50.49)	47 (42.34)	
5000-10000	21 (20.39)	30 (27.03)	
>10000	4 (3.88)	3 (2.70)	
Education[e]			
Primary	1 (0.97)	4 (3.60)	0.580
Some secondary	53 (51.46)	53 (47.75)	
Completed secondary	39 (37.86)	38 (34.23)	
Some Tertiary	6 (5.83)	11 (9.91)	
Completed Tertiary	4 (3.88)	5 (4.50)	
Employed[f]	27 (26.21)	39 (35.14)	0.242
Age Enrolment (years)	28.78 (5.75) [18.00 – 40.70]	29.12 (5.85) [19.00 – 42.60]	0.671
Anaemia during pregnancy[f]	29 (28.16)	27 (24.32)	0.753
Smoking during pregnancy[e]	4 (3.88)	6 (5.41)	0.750
Alcohol during pregnancy	9 (8.74)	4 (3.60)	0.199
Depression during pregnancy	17 (16.50)	22 (19.82)	0.652
HIV infection during pregnancy	38 (36.89)	39 (35.14)	0.900
**Infant Characteristics**			
Haemoglobin (g/dL)[g]	9.96 (0.72) [7.30 – 11.00]	11.42 (0.79) [9.50 – 14.10]	<0.001***
Age at Hb Measurement (Months)[g]	6.60 (4.09) [2.33 – 18.67]	7.40 (4.32) [2.01 – 18.05]	0.170
Sex (boys)	49 (47.57)	58 (52.25)	0.584
Gestational age at birth (weeks)[d,f]	38.80 (2.17) [30.00 – 42.00]	39.40 (1.35) [35.00 – 42.00]	0.034*
Child age at scan (months)[h]	11.50 (5.51) [3.12 – 23.92]	11.91 (5.58) [2.66 – 25.49]	0.487
HIV Infection[e,f]	1 (0.97)	0 (0.00)	0.463
Birth weight (g)[c,d,f]	3132.21 (559.55) [1180.00 – 4390.00]	3164.91 (440.02) [2160.00 – 4750.00]	0.635
Birth length (cm)[c,d,f]	49.31 (4.01) [33.00 – 57.00]	50.38 (2.81) [41.00 – 57.00]	0.027*
Birth head circumference (cm)[c,f]	34.33 (1.81) [28.00 – 38.00]	34.71 (1.54) [31.00 – 40.00]	0.108

Abbreviations: ULF, ultra-low-field; mT, millitesla; ZAR, South African Rand; Hb, haemoglobin; HIV, Human Immunodeficiency Virus; g, grams; cm, centimetres.

SI conversion factor: To convert to haemoglobin grams per litre, multiply by 10.

Group differences based on child anaemia status were calculated using unpaired *t*-tests for continuous data and chi-squared tests or Fisher's exact tests for categorical data.

[a] The total and group subsamples represent the number of unique subjects, excluding repeated measures data for children with multiple scans, to avoid duplication of observations for infants with multiple scans. Values for continuous variables are presented as: mean (standard deviation) [range]. Values for categorical variables are presented as: count (%).

[b] Dichotomous classifications for postnatal child anaemia at each study visit were determined using age-specific cut-offs for haemoglobin based on WHO guidelines for children over 6 months and local guidelines for children under 6 months. An overall classification for child anaemia status was determined based on the diagnosis of anaemia at least once across study visits. This was used for the assessment of group differences in sample characteristics between infants who had ever been anaemic versus infants who had never been anaemic between 3 and 24 months of age.

[c] The birth anthropometry were conducted by trained labour staff in the ward. Infant length was measures in cm to the nearest completed 0.5cm and weight was measured in kgs.

[d] Levene's test was significant. T-test results were interpreted based on equal variance not assumed.

[e] Fisher's exact test result interpreted due to one or more cells having an expected count of less than 5.

[f] Missing Values: Maternal employment (n = 2), monthly household income (n = 20), infant HIV infection (n = 147), maternal anaemia status (n = 37), child gestational age at birth (n = 43), infant birth weight (n = 2), infant birth length (n = 5), infant birth head circumference (n = 5).

[g] Minimum child haemoglobin and corresponding age were determined for each unique infant (n = 214) from data across study visits in the repeated measures subsample.

[h] Mean child age at scan was calculated for the full subsample of scans (n = 341) including repeated measures, given that each child may have had multiple scans at different ages from different study visits. The group summary statistics for child age at scan represent the mean, standard deviation, and range for each independent observation.

*p is significant at <0.05, **p is significant at <0.01, ***p is significant at <0.001.

Table S3. Maternal and infant characteristics for the ultra-low-field (ULF; 64 mT) subsample according to BRINDA-adjusted antenatal maternal iron deficiency status

Variable[a]	Total Subsample (n = 73)[a]		
	Maternal Iron Deficiency [b] (n = 29)	No Maternal Iron Deficiency [b] (n = 44)	p
**Maternal Characteristics**			
Adjusted serum ferritin (μg/L) [d]	11.02 (2.40) [6.25 – 4.81]	35.09 (23.36) [15.03 – 139.28]	<0.001***
Gestational age at serum ferritin measurement [f]	33.86 (3.31) [28.00 – 40.00]	31.81 (3.31) [27.00 – 38.00]	0.032*
Pregnancy trimester serum ferritin measured [e,f]			0.112
First	2 (6.90)	6 (13.64)	
Second	4 (13.79)	13 (29.55)	
Third	15 (51.72)	13 (29.55)	
Trimester unknown	8 (27.59)	12 (27.27)	
Monthly household income (ZAR)[e,f]			1
<1000	6 (20.69)	8 (18.18)	
1000-5000	13 (44.83)	19 (43.18)	
5000-10000	5 (17.24)	7 (15.90)	
>10000	0 (0.00)	0 (0.00)	
Education[e]			0.485
Primary	0 (0.00)	1 (2.27)	
Some secondary	13 (44.83)	19 (43.18)	
Completed secondary	15 (51.72)	18 (40.91)	
Some tertiary	0 (0.00)	4 (9.09)	
Completed tertiary	1 (3.45)	2 (4.55)	
Employed	10 (34.48)	12 (27.27)	0.692
Age at enrolment (years)	29.12 (5.59) [19.60 – 40.30]	28.59 (5.96) [19.00 – 39.40]	0.705
Smoking during pregnancy[e]	0 (0.00)	1 (2.27)	1
Alcohol during pregnancy[e]	0 (0.00)	3 (6.82)	0.272
Depression during pregnancy	8 (27.59)	8 (18.18)	0.508
HIV infection during pregnancy	14 (48.28)	15 (34.09)	0.333
**Infant Characteristics**			
Sex (male)	19 (65.52)	17 (38.64)	0.045*
Gestational age at birth (weeks)[d,f]	39.62 (1.02) [37.00 – 41.00]	38.69 (1.99) [33.00 – 41.00]	0.054
Child age at scan (months, n = 128) [g]	10.69 (5.09) [2.99 – 21.09]	10.48 (5.49) [2.69 – 25.07]	0.826
HIV infection [e,f]	0 (0.00)	(0.00)	N/A
Birth weight (g)[c,f]	3278.97 (448.66) [2400.00 – 4360.00]	3104.19 (508.62) [1780.00 – 4100.00]	0.139
Birth length (cm) [c,f]	50.52 (3.00) [44.00 – 57.00]	49.86 (3.33) [41.00 – 57.00]	0.395
Birth head circumference (cm) [c,d,f]	34.88 (1.31) [32.00 – 38.00]	34.32 (1.79) [30.50 – 37.00]	0.156

Abbreviations: ULF, ultra-low-field; mT, millitesla; μg/L, micrograms per litre; ZAR, South African Rand; HIV, Human Immunodeficiency Virus; g, grams; cm, centimetres

Group differences based on antenatal maternal iron deficiency status were calculated using unpaired *t*-tests for continuous data and chi-squared tests or Fisher's exact tests for categorical data.

[a] The total and group subsamples represent the number of unique subjects, excluding repeated measures data for children with multiple scans, to avoid duplication of observations for infants with multiple scans. Values for continuous variables are presented as: mean (standard deviation) [range]. Values for categorical variables are presented as: count (%).

[b] Serum ferritin concentrations were adjusted for inflammation using the BRINDA regression correction approach. Antenatal maternal iron deficiency classified as adjusted serum ferritin concentrations <15µg/L.

[c] The birth anthropometric measurements were conducted by trained labour staff in the ward. Infant length and head circumference were measured in cm to the nearest completed 0.5cm and weight was measured in kgs.

[d] Levene's test was significant. *T*-test results were interpreted based on equal variance not assumed.

[e] Fisher's exact test result interpreted due to one or more cells having an expected count of less than 5.

[f] Missing values: Trimester serum ferritin measured ($n = 20$), gestational age at serum ferritin measurement ($n = 20$), household income ($n = 15$), child gestational age at birth ($n = 20$), infant HIV ($n = 48$), infant birth weight ($n = 1$), infant birth length ($n = 2$), infant birth head circumference ($n = 2$).

[g] Mean child age at scan was calculated for the full subsample of scans ($n = 128$) including repeated measures, given that each child may have had multiple scans at different ages from different study visits. The group summary statistics for child age at scan represent the mean, standard deviation, and range for each independent observation.

**p* is significant at <0.05, ** *p* is significant at <0.01, ****p* is significant at <0.001.
